# Organic and inorganic nutrients modulate taxonomic diversity and trophic strategies of small eukaryotes in oligotrophic oceans

**DOI:** 10.1093/femsmc/xtac029

**Published:** 2022-12-07

**Authors:** Naomi Villiot, Amy E Maas, Alex J Poulton, Leocadio Blanco-Bercial

**Affiliations:** The Lyell Centre for Earth and Marine Science, Heriot-Watt University, Edinburgh, Scotland EH14 4AS, United Kingdom; Bermuda Institute of Ocean Sciences, 17 Biological Station, St George’s, GE01, Bermuda; The Lyell Centre for Earth and Marine Science, Heriot-Watt University, Edinburgh, Scotland EH14 4AS, United Kingdom; Bermuda Institute of Ocean Sciences, 17 Biological Station, St George’s, GE01, Bermuda

**Keywords:** marine protists, metabarcoding, nutrient competition, biodiversity, trophic strategy, dissolved inorganic phosphorus

## Abstract

As the oligotrophic gyres expand due to global warming, exacerbating resource limitation impacts on primary producers, predicting changes to microbial assemblages and productivity requires knowledge of the community response to nutrient availability. This study examines how organic and inorganic nutrients influence the taxonomic and trophic composition (18S metabarcoding) of small eukaryotic plankton communities (< 200 µm) within the euphotic zone of the oligotrophic Sargasso Sea. The study was conducted by means of field sampling of natural microbial communities and laboratory incubation of these communities under different nutrient regimes. Dissimilarity in community composition increased along a depth gradient, with a homogeneous protist community within the mixed layer and distinct microbial assemblages at different depths below the deep chlorophyll maximum. A nutrient enrichment assay revealed the potential of natural microbial communities to rapidly shift in composition in response to nutrient addition. Results highlighted the importance of inorganic phosphorus availability, largely understudied compared to nitrogen, in constraining microbial diversity. Dissolved organic matter addition led to a loss of diversity, benefiting a limited number of phagotrophic and mixotrophic taxa. Nutrient history of the community sets the physiological responsiveness of the eukaryotic community to changing nutrient regimes and needs to be considered in future studies.

## Teaser

The incubation of natural microbial communities with different nutrient regimes revealed the importance of inorganic phosphorus as a controller of biodiversity in oligotrophic waters, indicated a strong coupling of the eukaryotic community taxonomy and trophic strategy, and lead to a reduction in diversity in favour of heterotrophic assemblages following the addition of dissolved organic matter.

## Introduction

In marine ecosystems, planktonic assemblages include a large diversity of unicellular eukaryotes (protists), small zooplankton, and fungi (Tragin and Vaulot 2018, Wu et al. 2020). Protists are diverse in terms of both their taxonomy and trophic modes (Caron et al. 2008, Caron et al. 2012), and include primary producers (photosynthetic protists), consumers (heterotrophic protists), parasites and saprotrophs (Keck et al. 2020). Photosynthetic protists, such as diatoms, dinoflagellates, and haptophytes account for a relatively large fraction of net primary production (Duarte and Cebrián 1996). Mixotrophic taxa in each of these protist groups (Havskum and Riemman 1996) have the ability to live as both photoautotrophic primary producers and as heterotrophic consumers through ingestion and digestion of organic particles (Sanders 1991). In the last decade, mixotrophy has been recognised as an important nutritional route for plankton communities (Leles et al. 2019, Schneider et al. 2020). For example, mixotrophic flagellate grazing on bacteria can contribute 50% to 95% of bacterial mortality (Unrein et al. 2007, Zubkov and Tarran 2008, Anderson et al. 2018), and significantly impact trophic transfer efficiency and the biological carbon pump (Ward and Follows 2016).

While changes in the taxonomic composition of photosynthetic primary producers, both strict photoautotrophs and mixotrophs, have an impact on higher trophic levels and ecosystem processes (Payne 2013), the ecology and distribution of many protists in the ocean remains poorly known. In addition, heterotrophic eukaryotes (in this study phagotrophs) such as ciliates and some dinoflagellates, are key component of food-webs as they act as important microbial predators consuming bacteria and small protists, and ultimately are preyed upon by mesozooplankton (Sherr and Sherr 2002, Sherr et al. 2007). Consequently, compositional changes of planktonic protists are sensitive to both bottom-up controls through nutrient-limitation and food-supply, and top-down controls through zooplankton predation and mortality (Payne 2013).

The composition of plankton communities is controlled by both biotic factors (food availability, grazing mortality, bacterial and viral infections) and abiotic factors (light and nutrient availability, temperature) (Sherr et al. 2007, Caron et al. 2016). As individual taxa vary in their response to environmental factors, understanding protist diversity and taxa-specific interactions with the environment is key to elucidating the ecological processes involved in controlling the structure and functionality of planktonic ecosystems (Naeem and Li 1997, Wu et al. 2020). In warm subtropical waters, plankton communities exist in a highly-stratified ecological environment, with (inorganic) nutrient limitation in sun-lit upper waters and light limitation at depth where inorganic nutrients are replete (Sigman and Hain 2012).

This study examines the following predictions of resource competition: (i) changes in inorganic nutrients, particularly nitrogen (N), will lead to significant changes in community taxonomic and trophic composition, (ii) labile dissolved organic N (DON) and dissolved organic phosphorus (DOP) availability will benefit photosynthetic protist growth, and (iii) increased nutrient availability will lead to the dominance of a limited number of taxa.

N and phosphorus (P) are the primary limiting nutrients for the growth of photosynthetic protists, with N and P availability regulating primary production and community structure (Moore et al. 2013). In fact, protists and other marine microbes are the main drivers of the global cycling of carbon, N and P in the ocean (Arrigo 2005), through N and P uptake during growth and their loss to the dissolved phase through mortality or to depth via the biological carbon pump (Lalli and Parsons 1997).

Studies demonstrating N limitation of protist growth in the oligotrophic ocean are numerous (e.g. Ryther and Dunstan 1971, Mills et al. 2004), with phytoplankton biomass and productivity often increased only after the addition of N (e.g. Graziano et al. 1996, Moore et al. 2008). Due to the low atmospheric input of P into the ocean (Broecker and Peng 1982), P may be an additional nutrient controlling primary production and the biological carbon pump (Moore et al. 2013). Evidence of P stress has been reported in microbial communities, with studies emphasizing P as a limiting nutrient on physiologically-relevant time scales in the oligotrophic North Atlantic (e.g. Wu et al. 2000, Lomas et al. 2004, Van Mooy et al. 2009) and eastern Mediterranean (e.g. Thingstad et al. 2005).

Furthermore, although nutrient cycling studies often focus on inorganic N and P, in oligotrophic waters DOP may account for up to ∼ 80% of total dissolved P (Ruttenberg and Dyhrman 2005) and DON may account for up to ∼ 65% of total dissolved N (Bronk 2002). The remineralisation of DON and DOP in oligotrophic waters is thought to be mainly by heterotrophic microbes (Berman and Bronk 2003), however, the use of DON and DOP by photosynthetic protists has also been widely reported (e.g. Antia et al. 1991, Mulholland and Lomas 2008, Moschonas et al. 2017), and allows such primary producers to adapt to inorganic P limitation and support their continued growth (Fitzsimons et al. 2020).

Experimental addition of inorganic nutrients has allowed the assessment of nutrient limitation on protist growth (e.g. Hunt and Matveev 2005, Khairul and Rahanim 2015), and physiology and photosynthesis (e.g. Menzel and Rhyther 1961, Graziano et al. 1996, Moore et al. 2008). In contrast, few studies have used microscopy to determine the effect of inorganic nutrients on community diversity and taxonomic interactions (e.g. Hunt and Matveev 2005, Burson et al. 2018). Such studies are often restricted to a limited number of taxa, including cyanobacteria and protists identifiable by microscopy or flow cytometry (e.g. Graziano et al. 1996, Moore et al. 2008), though this cannot account for the full diversity of protists (Li 1994, 2002). A failure to fully assess changes in taxonomic or functional diversity of the community ignores how species composition drives primary production and impacts the biological carbon pump. The small ribosomal subunit 18S gene and the region V4 are commonly used in metabarcoding studies, and can be used to determine the community diversity and species dynamics of aquatic eukaryotic protists (Martin et al. 2022).

In this field study, we examined the phylogenetic and trophic structure of protist communities in the Sargasso Sea during the fall of 2020 as well as the changes in composition triggered by addition of nutrients. Natural plankton communities were sampled and incubated during a nutrient enrichment assay to assess the effect of inorganic and organic nutrient availability on protist taxonomic and functional composition by means of 18S V4 DNA-metabarcoding.

## Method

### Study site and sampling of the natural community

Natural plankton communities were sampled within the Bermuda Atlantic Time-series Study (BATS) program at Hydrostation ‘S’ (coordinates 32°10’N, 64°3’W) in the Sargasso Sea (Atlantic Ocean) during research cruises aboard the *R.V. Atlantic Explorer* in the Fall of 2020 (see Supplementary Table S1 for sampling dates, times, and depths). Seawater samples were obtained using Niskin bottles (14 L) mounted on a rosette sampler equipped with a conductivity, temperature, and depth (CTD) sensor (Sea-Bird Electronics, see http://bats.bios.edu for details of the models of sensors). Physical and chemical data were accessed via the BATS ftp repository (http://batsftp.bios.edu). Specific details for all the methods can be found in Knap et al. (1997), though a brief description is given below.

Samples for nucleic acid extraction were collected by filtering 1 L through a 0.22 μm pore size nitrocellulose membrane filtration (47 mm diameter, Millipore Corp.). Filters were placed into sterile Eppendorfs in single cell lysis buffer (SLB; 40 mM EDTA, 50 mM Tris-HCl, 0.75 M sucrose) and kept at −20°C until extraction. Samples for chlorophyll-*a* (Chl-*a*) were collected by filtering 250 mL through glass fibre filters (GF/F, 25 mm diameter, Whatman®). Filters were placed into centrifuge tubes and stored at −20°C until analysis. Samples for inorganic nutrient analysis were collected in 50 mL acid-washed centrifuge tubes and stored at −20°C until analysis. For flow cytometry, 2 mL samples were placed into sterile Eppendorf, fixed with glutaraldehyde (0.25% final concentration) and stored at −20°C in the dark until analysis.

### Experimental setup of the nutrient enrichment assay

The experiments were conducted in a climate-controlled room allowing full control of light and temperature conditions. The working volume of the incubation bottles was 2.2 L. Incubation bottles were filled with seawater from Hydrostation ‘S’ passed through a 200 µm mesh to exclude large meso-zooplankton (> 200 µm), whose low abundance (∼ 0.15–0.35 individuals L^−1^; Maas et al. 2021) would make their inclusion statistically rare. Nutrient enrichment assays were initiated by placing bottles from different depths into their respective light/temperature incubation conditions (Table S1) and by adding different inorganic and organic nutrient sources of nitrogen (N) and phosphorus (P). Light intensities and temperatures were comparable to those measured at initial sampling depths during the research cruise (Table S1). Incubation bottles were incubated for 4 days under a 12:12 light/dark cycle (L/D).

The N and P treatments were: control, no addition; addition of dissolved inorganic nitrogen (DIN); addition of dissolved inorganic phosphorus (DIP); addition of dissolved organic nitrogen (DON) and dissolved organic phosphorus (DOP) (see Supplementary Table S2 for further details). Treatments with N sources had concentrations of ∼ 2 µmol N L^−1^ added and treatments with P sources had concentrations of ∼ 0.2 µmol P L^−1^ added. The DON components (Table S2) comprised of protein-forming amino acids found in natural marine DON (Antia et al. 1991), with urea added to prevent nitrogen-nickel co-limitation (Price and Morel 1991), and DOP comprised of adenosine mono- and tri-phosphate (AMP and ATP, respectively), as well as glycerophosphate and β-nicotinamide adenine dinucleotide (NAD), which are all representatives of natural marine DOP (Benitez- Nelson, 2000). ATP is a labile form of DOP, in the low molecular weight fraction (< 10 kDa); with uptake of ATP studied in marine systems and reports of a preference for this DOP source in recent studies (Diaz et al. 2018, Nausch et al. 2018).

### Inorganic nutrients and chlorophyll-a

Samples for nitrate (NO_3_) + nitrite (NO_2_), soluble reactive phosphorus (SRP), and silicic acid (Si(OH)_4_) were analysed on a Technicon AutoAnalyzer II with an analytical precision of approximately 0.03 to 0.05 µmol L^−1^ for all nutrients. Chl-*a* concentrations were determined by fluorometric analysis following Knap et al. (1997). Filters were placed in 100% acetone and stored at −20°C in the dark overnight. Samples were centrifuged at 1000 × *g* for 5 min at room temperature. Supernatants were place into glass test tubes with 90% acetone. Samples were then analysed using a Turner fluorometer before and after acidification with 1 M HCl (Knap et al. 1997).

### DNA extraction and sequencing

DNA was extracted from the samples using the method described by Giovannoni et al. (1990) with the following adjustments: lysozyme and RNAse were not added as they lower DNA purity (Fuhrman et al. 1988). Nucleic acid extraction began with the addition of proteinase K (Sigma-Aldrich; St. Louis, MO, United States) at a final concentration of 0.2 mg mL^−1^ and sodium dodecyl sulfate (SDS) (1%), and then filters were incubated at 37°C for 30 minutes and 55°C for 1 hour. The lysates (∼ 1 mL) were recovered from the filters and transferred to fresh sterile Eppendorf tubes. The pooled lysates were then extracted once with phenol-chloroform-isoamyl alcohol (25:24:1; pH 8) and twice with chloroform-isoamyl alcohol (24:1). The cesium trifluoroacetate purification steps were not required in this process. The aqueous phase was then placed into fresh sterile Eppendorf tubes and the DNA was purified by precipitation using 3 M Sodium Acetate and 100% isopropanol (sample: isopropanol 1:1). Extraction tubes were stored at −20°C overnight. Samples were concentrated by 80% ethanol precipitation, vortexed for 30 s and centrifuged at 20 000 xg for 10 min. The resulting pellets were dried at 37°C and stored at −20°C until analysis.

Amplification and sequencing were carried out at the Center for Genome Research and Biocomputing at Oregon State University. The 18S V4 region was amplified using the primers V4 forward 5-CCAGCA[GC]C[CT]GCGGTAATTCC-3 and Reverse 5-ACTTTCGTTCTTGAT[CT][AG]A-3 (Stoeck et al. 2010), using the KAPA HiFi HotStart ReadyMix (Kapa Biosystems) following manufacturer protocols. The PCR thermal cycler program consisted of a 98°C denaturation step for 30 s, 10 cycles of 10 s at 98°C, 30 s at 53°C and 30 s at 72°C, then 15 cycles of 10 s at 98°C, 30 s at 48°C, and 30 s at 72°C, followed by a final elongation step at 72°C for 10 min. After the first PCR, dual indices and Illumina sequencing adapters were attached using the Illumina Nextera XT Index Kit, following manufacturer protocols for amplicon library preparation (Part # 15044223 Rev. B). Amplicon sequencing was conducted on an Illumina MiSeq using V2 chemistry and 2×250 paired end procedure, aiming for an average of 75 000 reads per sample.

### Bioinformatics pipeline

Bioinformatics followed Blanco-Bercial et al. (2022). Quality control and read merging were carried out with MeFiT (Parikh et al. 2016) with very strict parameters: CASPER (Kwon et al. 2014), K-mer size of 5, threshold for difference of quality score 5, threshold for mismatching ratio 0.1, a minimum length of overlap of 15, and using Jellyfish Ver. 2 (Marçais and Kingsford 2011). The merge and quality filter parameters (using the *meep* score; Koparde et al. 2017) were set at a filtering threshold of 0.1 and discarding non-overlapping reads. Assembled reads passing the quality control were duplicated and complementary/reversed to be fed into MOTHUR Ver. 1.43 (Schloss et al. 2009), using the script available at the GitHub repository: https://github.com/blancobercial/Protist_Time_Series. In short, after trimming to the V4 region (by aligning to the SILVA 138 release trimmed to the V4 region) and removal of chimeras with VSEARCH (Rognes et al. 2016), single variants were obtained using UNOISE2 (Edgar 2016) as implemented in MOTHUR, with diffs = 1. Taxonomic assignment was done using the PR2 database (version 4.14.0; Guillou et al. 2013), a database suited for protists. After excluding metazoan and ‘Eukaryota_unclassified’ and standardization through rarefaction of all samples to 50 000 reads, all amplicon sequence variants (ASVs) with less than two reads (equivalent to global singletons) were equal to zero.

### Phytoplankton flow cytometry analysis

Fixed seawater samples were initially thawed at room temperature in the dark, filtered through nylon-mesh filters with 50 μm pore size (CellTrics®, Sysmex Partec), supplemented with 20 µL of a flow check high intensity bead solution of 3 μm diameter beads (Polysciences, USA), and then homogenized with a vortex for few seconds. The flow cytometer (Partec Cube 8, Sysmex Partec GmbH, Goerlitz, Germany) was equipped with a blue (488 nm) and red laser (640 nm). Cells were characterized by using scatter and fluorescence signals collected from the 488 nm laser as ‘cyanobacteria’ (including both *Prochlorococcus* and *Synechococcus)* and ‘eukaryotes’ (corresponding to photosynthetic eukaryotes with cell diameters > 2 µm). The forward light scatter (FSC) and the side scatter (SSC) were related to the cell size, structure, and shape, respectively. Orange (FL2, 590 ± 50 nm) and red (FL3, 675 ± 25 nm) fluorescence signals corresponded to the presence of phycoerythrin and Chl-*a*, respectively. Red fluorescence (FL3) was used as the trigger signal. Flow rate was 2 µL s^−1^ and volume for analysis was 800 µL. Mono-cultures of the haptophyte *Tisochrysis* sp. (cell diameter of ∼ 3 µm) were used for fluorescence and size calibration. Data were analysed with the FCS Express 5 software (De Novo).

### DNA-barcoding dataset and taxonomic and trophic attributions

After the filtering (removal of singletons, as well as low count amplicon sequence variants (ASVs) and processing of the raw data (see section 6)), a total of 2 889 031 DNA reads were retained and clustered into 3731 ASVs. ASVs were classified into defined taxonomic ranks of the Adl et al. (2019) classification. Around 98.66% of the ASVs were classed at the 2^nd^ rank level into 19 groups and ∼ 95.98% of the ASVs were classed at the 3^rd^ rank level into 42 groups. Over 77.46% of the ASVs were assigned to one of five trophic groups (i.e. mixotrophs, parasites, phagotrophs, photoautotrophs, saprotrophs), the rest of the ASVs (841) were assigned to the group ‘trophy undetermined’. Importantly, deviation in the number of gene copies across plankton taxa (Not et al. 2009) can significantly affect the proportion of reads per taxa in complex microbial assemblages and lead to misinterpretation of relative abundances (Santi et al. 2021). This is particularly true in the case of Dinophyceae which tend to have a higher copy number of 18S rDNA than most lineages (Martin et al. 2022). As Dinophyceae taxa made up a large proportion of our dataset, the Dinophyceae that could not be classified as phagotrophs, mixotrophs, or photoautotrophs (91.91% of unclassified ASVs) were clustered as a separate functional group, ‘unclassified Dinophyceae’. Finally, all taxa with less than 10 reads were dropped from the analysis as well as the taxa attributed to the trophic groups ‘parasites’ and ‘saprotrophs’ (see Supplementary text 1 for details of the ASV richness and total reads for each trophic group) which left a total of 2014434 DNA reads and these were clustered into 2164 ASVs.

### Statistical analysis

Data were manipulated and transformed in R (tidyverse, dplyr, phyloseq, taxa, metabarcoder, and microbiome). To control for differences in sequencing between samples, count data were converted into relative proportions. The analysis of the α-diversity (Shannon measure) was performed to determine how diverse the communities were (R, package phyloseq) and was followed by the subsequent tests: Kruskal-Wallis rank sum test and Dunn's Kruskal-Wallis multiple comparison with *P*-values adjusted with the Benjamini-Hochberg method (R, package FSA) performed in groups of samples for which significant differences were identified by the Kruskal-Wallis test. Analysis of the β-diversity was performed to assess how different the communities were. Data were first transformed (Hellinger square root transformation), then a multivariate analysis was applied to assess the (dis)similarity in ASV composition of samples using Bray-Curtis similarity and non-metric multidimensional scaling (nMDS) ordination (R, package vegan).

Significance of the observations from the β-diversity were assessed by: (i) an analysis of similarities (ANOSIM) to statistically test for differences in eukaryote communities between two or more groups of samples; (ii) an analysis of the homogeneity of multivariate dispersion to assess whether the changes were due to the differences between treatments or the dispersion within groups of samples; (iii) a permutational multivariate analysis of the variance (PERMANOVA) assessing the significance of the difference between groups of samples due to the experimental treatments in terms of the Bray-Curtis dissimilarities (R, package vegan), as well as a pairwise comparison between groups with corrections for multiple testing (R, package RVAideMemoire) to identify the treatments responsible for the dissimilarity; and (iv) an analysis of the similarity (SIMPER) to estimate the contribution of each ASV (%) to the dissimilarity between each pair of treatments. Finally, a differential abundance (DESeq2) analysis on the non-transformed absolute counts was applied to identify the significant changes in reads between taxonomic and trophic groups (R, package DESeq2). All data were plotted in R (R, packages fantaxtic for barplots, ggplot2, and ggpubr).

## Results

### Vertical profiles at the sampling site

**Figure 1.**
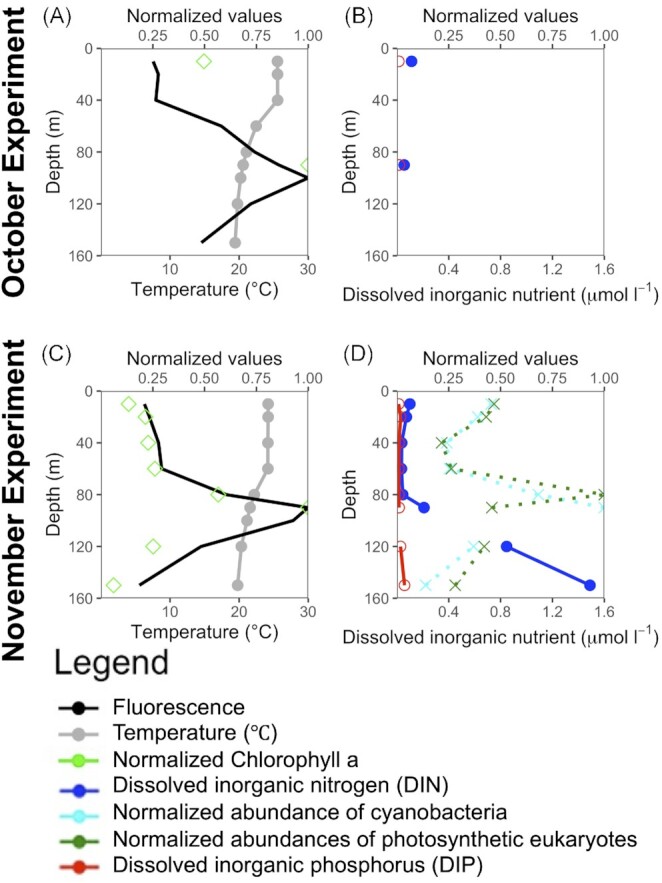
Vertical profiles of the physico-chemical and biological parameters at Hydrostation ‘S’ during sampling in October (**A, B**) and November (**C, D**). Temperature (grey dots) and fluorescence (solid black line) data correspond to *in situ* measurements recorded with a CTD. Normalized Chl-*a* (green diamonds), dissolved inorganic nitrogen (blue dots) and dissolved organic phosphorus (red dots), and normalized abundances of cyanobacteria (cross shape with dotted cyan line) and photosynthetic eukaryotes (cross shape with dotted green line) detected by flow cytometry (in November only), were all computed from samples collected with a rosette and processed in the laboratory.

The temperature profiles from late October (Fig. 1A) indicated a mixed layer depth of ∼ 40 m, while the mixed layer in mid-November extended to ∼ 60 m (Fig. 1C). In addition, both fluorescence from the CTD and discrete Chl-*a* profiles showed a shallowing of the deep chlorophyll maximum (DCM) from ∼ 100 m in late October to ∼ 90 m in mid-November (Fig. 1A, C). DIN and DIP concentrations were higher in the deep chlorophyll maximum than in the surface mixed layer (Fig. 1B, D). In November, the nutricline depth, defined as the depth where the nitrate concentration first becomes more than 0.05 μmol N L^−1^, was ∼ 80 m.

Both Chl-*a* concentration and fluorescence were found in higher quantities in the deeper part of the water column than the surface mixed layer, supporting higher biomass and/or cell pigmentation near the DCM (Fig. 1A, C). Peaks of fluorescence and Chl-*a* concentration corresponded to the highest abundance of cyanobacteria found in the depth profiles, with a cyanobacteria maxima at ∼ 90 m, while small photosynthetic eukaryotes (< 5 $\mu $m) were more abundant at ∼ 80 m in November (Fig. 1C, D).

**Figure 2.**
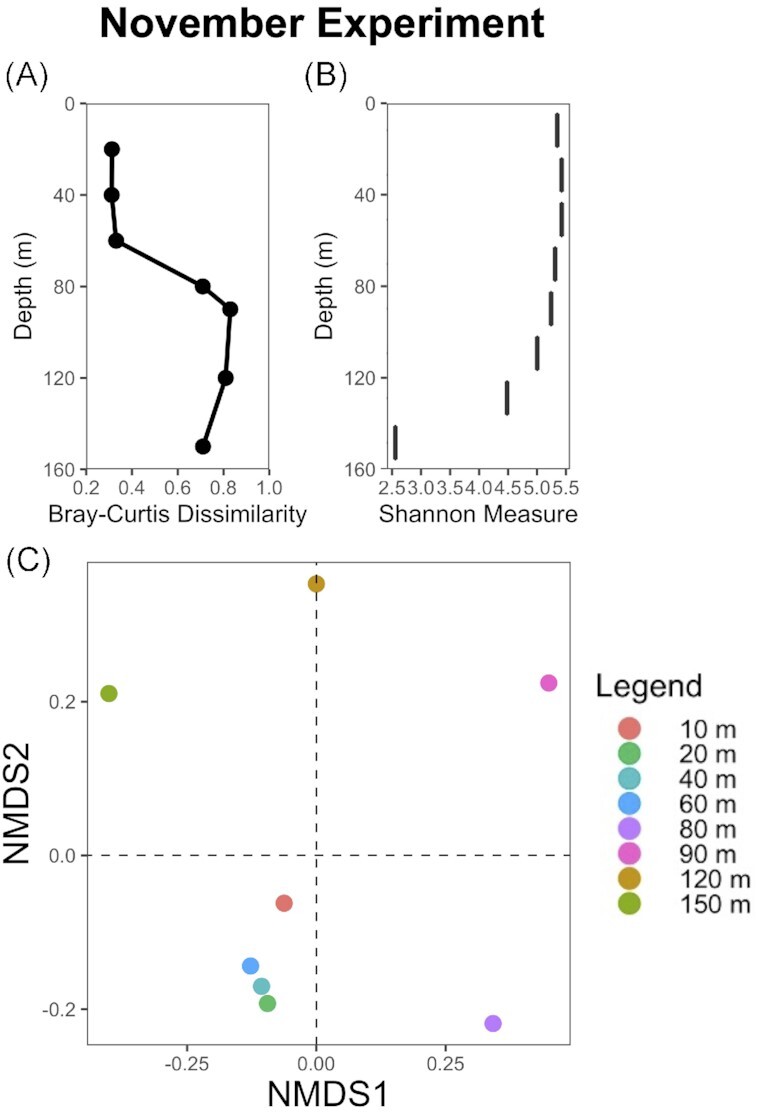
Analysis of the ASVs community composition along the depth gradient in November 2020: vertical profile of Bray–Curtis dissimilarity (**A**) and Shannon measures diversity (**B**), as well as non-metric multidimensional scaling (nMDS) ordination of the samples ASVs composition (**C**). Two-D stress indicated good fits (Kruskal, 1964) in the nMDS ordination, with a value less than 0.1.

In terms of community composition, the surface layer (10 m) and the deep layer (90 m) in October showed a strong dissimilarity of ∼ 70% between one another. In November, Bray-Curtis dissimilarity showed that the communities at 20 m, 40 m and 60 m (within the upper mixed layer) were compositionally similar, with dissimilarity less than ∼ 32% compared to the surface (10 m) (Fig. 2A, Supplementary Table S3). The depths below (80 m, 90 m, 120 m, 150 m) revealed strong compositional changes when compared to the surface layer, with dissimilarity ranging from 71 to 83% compared to 10 m (Fig. 2A, Supplementary Table S3).

Dissimilarity in community composition increased along the depth gradient with strong differences between each pair of depths at depths below 60 m, with dissimilarity ranging from ∼ 54% to 85% (Table S3). In contrast, dissimilarity between each pair of depths from the surface layer to 60 m was only ∼ 30% (Table S3). Shannon diversity indices for each depth sampled in November further demonstrated relatively similar values within the mixed layer (∼ 60 m), indicating communities of similar levels of diversity, and community differentiation below the deep layer (80 m) (Fig. 2B). Analysis of the β-diversities, based on Bray-Curtis dissimilarities of the relative abundances of ASVs, agreed with the assessment of Shannon diversity. The nMDS ordinations displayed noticeable differences between the clustering of samples from the mixed layer (i.e. depths 10 m; 20 m; 40 m; 60 m) and distinct clusters for each depth sampled below the deep layer, indicative of strong shifts in community ASVs (Fig. 2C). The ASVs detected in the samples also changed strongly across this vertical gradient. Of the total number of taxa present (693 ASVs), only 21 ASVs (∼ 3%) were found at all depths sampled in November, and only 29 ASVs (∼ 4%) were present at all depths below 80 m, whereas 204 ASVs (∼ 29%) were detected in all the mixed layer samples.

### Vertical ASV distribution and initial community composition

**Figure 3.**
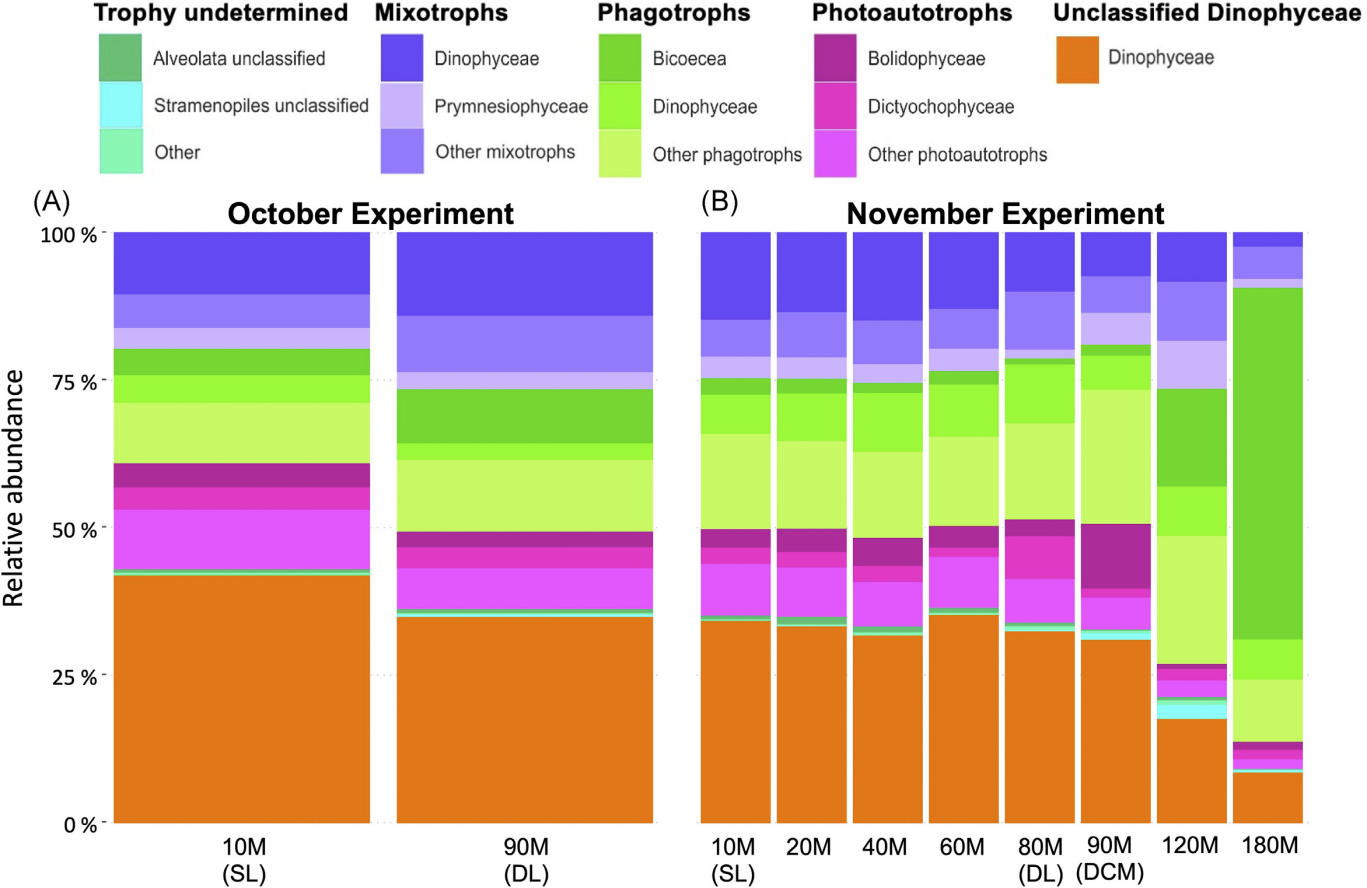
Taxonomic and functional composition (relative abundance, %) of the eukaryotic communities (excluding parasites and saprotrophs) in samples collected from different depths in the water column during October (**A**) and November (**B**). The two most abundant taxonomic classes are displayed for each functional group. Surface layers (SL), deep layers (DL) and deep chlorophyll maximum (DCM) are indicated.

Relative abundances of the different trophic groups (mixotrophs, unclassified Dinophyceae, phagotrophs, photoautotrophs, trophy undetermined) of the natural protist communities above the deep chlorophyll maximum, including both sampling depths (surface and deep layer), showed relatively similar compositions (Fig. 3A, B). Further details of the vertical distribution of taxonomic classes for each phylum in October and November 2020 are given in the (see Figs S1 and S2). Relative abundances based on the contribution of amplicon reads of trophic groups to total community amplicon reads, revealed that: below the deep chlorophyll maximum (> 90 m), the relative abundance of phagotrophs increased noticeably (Fig. 3B; Table S4). In October, the trophic composition of the protist community in both the surface and deep layers was similar (Fig. 3A; Table S4): ‘unclassified Dinophyceae’ (geometric mean 38.2% $\pm \,\,$ 4.6%) dominated, followed by mixotrophs (22.7% $\pm \,\,$ 4.0%; two most abundant taxonomic classes Dinophyceae and Prymnesiophyceae), phagotrophs (22.0% $\pm \,\,$ 3.6%; two most abundant taxonomic classes Bicoecea and Dinophyceae), photoautotrophs (15.1% $\pm \,\,$ 3.3%; two most abundant taxonomic classes Bolidophyceae and Dictyochophyceae) and ‘trophy undetermined’ (1.3% $\pm \,\,$ 0.3%; including mainly Alveolata and Stramenopiles classes). In November, in both the surface and deep layer and for all depths above the deep chlorophyll maximum, the major trophic group was phagotrophs (vertical profile geometric mean 32.8% $\pm \,\,$ 18.2%), followed by ‘unclassified Dinophyceae’ (25.6% $\pm \,\,$ 9.6%), mixotrophs (21.0% $\pm $ 5.6%), photoautotrophs (11.8% $\pm \,\,$ 5.1%) and ‘trophy undetermined’ (1.4% $\pm \,\,$ 0.9%). Within the DCM, relative abundances of ‘unclassified Dinophyceae’ and phagotrophs were similar (30.9% and 30.3%, respectively), while below phagotrophs became the most abundant trophic group and accounted for about half of the reads at 120 m and up to 77.0% of the reads at 150 m (Fig. 3B; Table S4).

Taxonomic distribution of the eukaryote community showed very similar vertical composition profiles between the depths above the DCM, with changes occurring from the deep chlorophyll maximum to the depths below (Fig. 3; Table S4). Relative abundances based on the contribution of amplicon reads of ASVs to total community amplicon reads, revealed that: Dinophyceae, including mixotrophic, phagotrophic and unclassified ASVs, were the most abundant at the time of sampling (t0) for both time periods, representing around half of the community amplicon reads (geometric mean 48.3% $\pm $ standard deviation 14.4%) (Fig. 3; Table S4). Non-photosynthetic stramenopiles were the next most abundant group, including the main phagotrophic classes Bicoecea, MAST, and Labyrinthulea, which all together accounted for 10.3% $\pm $ 19.2% of amplicon reads (Table S4). Stramenopile algae were the third most abundant group, accounting for 8.9 $\pm $ 4.0%, including the main classes Bolidophyceae (3.1% $\pm \,\,$2.8%), Dictyochophyceae (2.5% $\pm \,\,$1.7%), Pelagophyceae (1.1% $\pm $ 0.6%), Chrysophyceae (0.6% $\pm $ 0.2%) and Bacillariophyta (0.6% $\pm $ 0.4%) (Table S4). Retaria were the next most abundant group, contributing to 7.9% $\pm $ 1.7% of the amplicon reads (Table S4). Other abundant groups were the Ciliophora (3.9% $\pm $ 1.4%), Haptophyta dominated by the mixotrophic Prymnesiophyceae (3.6% ± 1.9%), Cryptista (2.5% $\pm $ 1.1%), and Chloroplastida (1.1% $\pm \,\,$ 0.5%) (Table S4). Further details of the vertical distribution of photosynthetic and phagotrophic taxa at Hydrostation ‘S’ are given in the Supplementary text 2.

### Bottle effects in the incubations

A comparison between the communities during sample collection (t0) from the surface and deep chlorophyll maximum with the controls after incubation was performed to assess potential ‘bottle effects’. The geometric mean of amplicon reads (ASVs) of all samples was 30782 reads (± 10 298). Examination of the number of ASVs in the samples indicated a loss of diversity in the samples after incubation (i.e. t0 versus controls). Specifically, a ∼ 78% loss in ASV richness between t0 and controls in October and a ∼ 59% loss in November. However, Bray–Curtis dissimilarity showed that the communities were compositionally similar between replicates (*n* = 3), with dissimilarities only around ∼ 33% (see Fig. S3, Table S5 for the month of October 2020, and Table S6 for the month of November 2020), while communities were compositionally different between t0s and controls with dissimilarities around ∼ 81% (Tables S5 and S6). The loss in ASV richness occurred across all trophic groups (Table 1). Overall, the relative abundance of ASVs detected by trophic groups between t0 and controls were relatively similar, apart from the group ‘unclassified Dinophyceae’, which decreased slightly in relative proportion in all controls (Table 1).

**Table 1.** Comparison of the ASV richness in percentages (%) per trophic group between natural communities (t0s) and communities after incubation (controls) in both the surface layer (SL) and deep layer (DL).

	October				November			
Trophic group	SL t0	SL control	DL t0	DL control	SL t0	SL control	DL t0	DL control
Mixotrophs	20.6 (±0.6)	20.0 (±1.5)	23.4 (±0.7)	28.4 (±2.3)	20.9	24.8 (±0.4)	25.5	25.0 (±4.2)
Unclassified Dinophyceae	37.6 (±1.0)	34.0 (±2.9)	36.8 (±1.3)	28.1 (±2.4)	36.7	34.5 (±1.8)	32.6	29.1 (±4.0)
Phagotrophs	24.0 (±0.3)	26.4 (±4.7)	22.8 (±1.2)	25.0 (±2.5)	26.6	27.1 (±1.1)	24.6	26.4 (±0.3)
Photoautotrophs	14.9 (±0.3)	17.8 (±1.4)	14.3 (±0.4)	15.7 (±2.1)	13.5	13.2 (±0.7)	14.4	17.9 (±1.2)
Trophy undetermined	3.0 (±0.5)	1.8 (±0.6)	2.7 (±0.2)	2.9 (±0.8)	2.3	0.4 (±0.4)	2.9	1.6 (±0.9)

**Figure 4.**
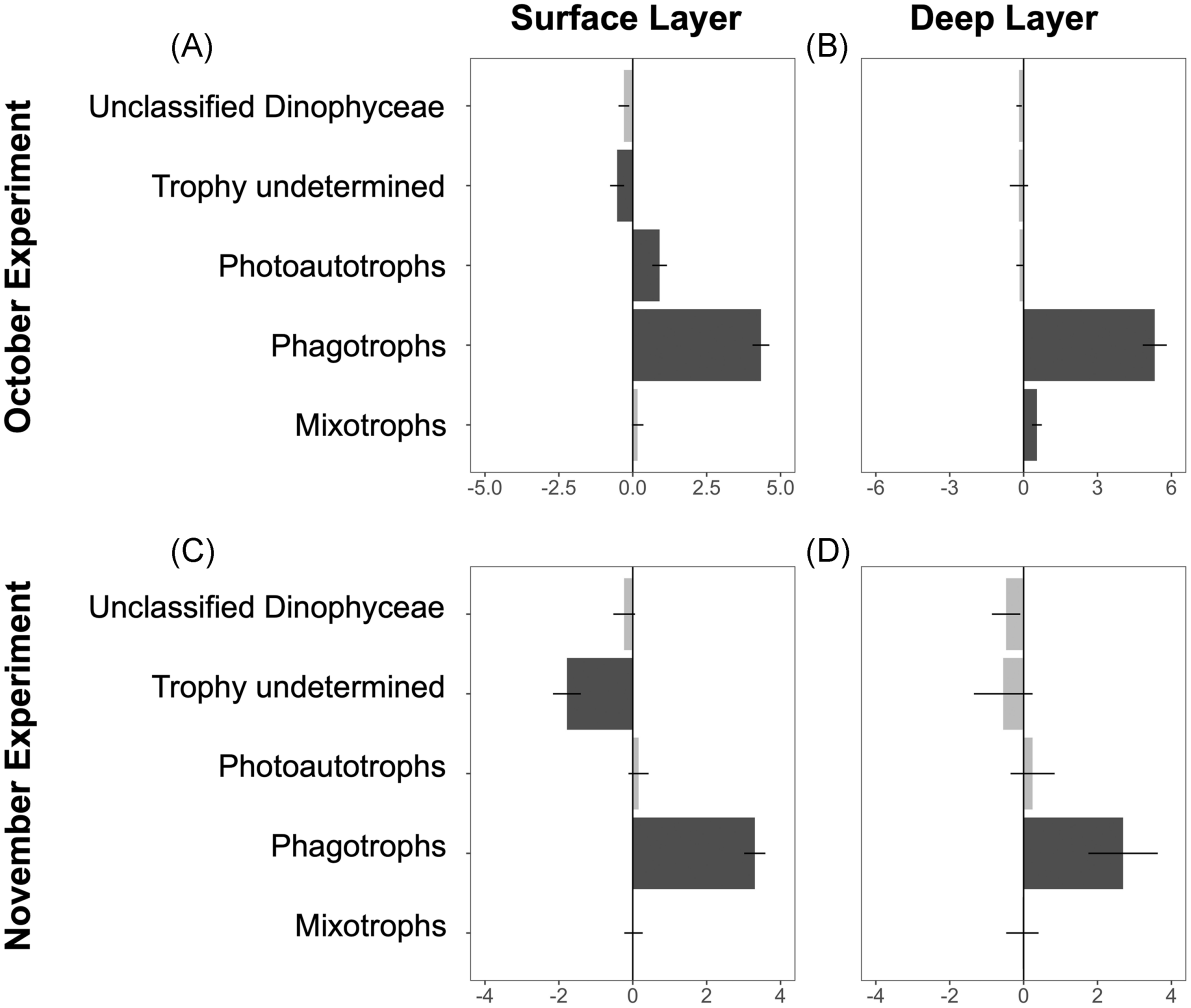
Magnitude of change in trophic groups between initials (t0) and controls for the month of October (**A, B**) and November (**C, D**). Magnitude of change is expressed in log2 fold change, as estimated by the DESeq2 analysis. Dark bars represent groups for which the change was statistically significant (*P*-values < 0.05, two-sided Wald test corrected with the Benjamini & Hochberg method). Horizontal lines show the standard error.

Differential abundance (DESeq2) analysis showed that the ‘bottle effects’ were generally consistent between the surface and deep layer communities for both October (Fig. 4A, B) and November (Fig. 4C, D) in term of trophic composition. The relative abundances of the trophic group ‘photoautotrophs’ did not significantly change, except for a slight increase in the surface layer in October (Fig. 4A). In contrast, the relative abundances of the trophic group ‘phagotrophs’ significantly increased in all comparisons (Fig. 4). Relative abundances of protists with ‘trophy undetermined’ decreased in the surface layers during both experiments (Fig. 4A, C), with ‘mixotrophs’ slightly higher in the deep layer during the experiment in October (Fig. 4B).

**Figure 5.**
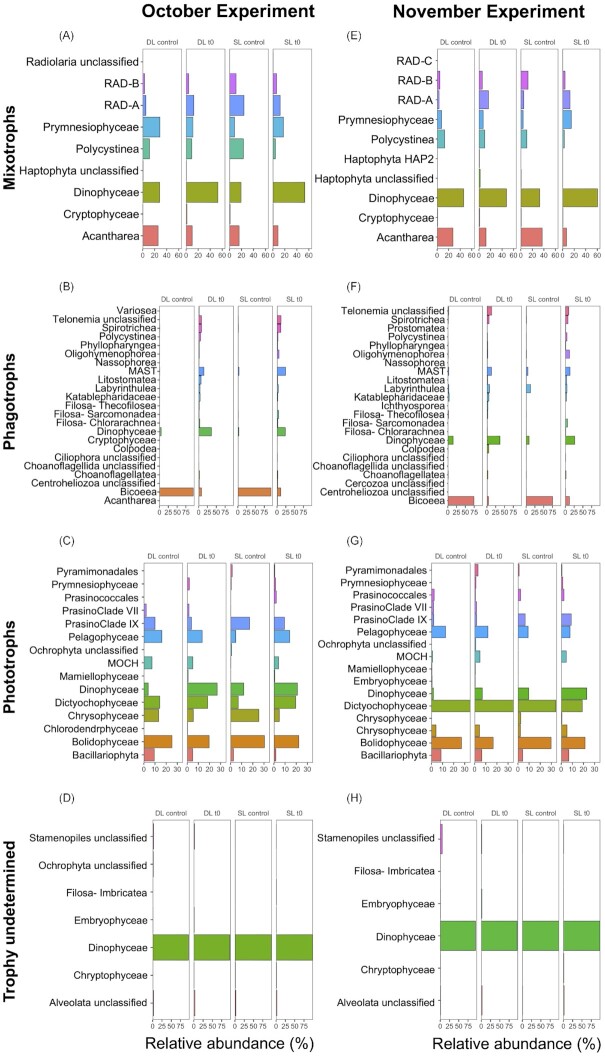
Relative abundances of the taxonomic classes within the functional groups mixotrophs (**A, E**), phagotrophs (**B, F**), photoautotrophs (**C, G**), and ‘trophy undetermined’ including the ‘unclassified Dynophyceae’ (**D, H**) between sampling times (t0s) and controls from the surface layer (SL) and deep layer (DL) in October 2020 (**A, B, C, D**) and November (**E, F, G, H**) 2020.

Importantly, the ASV community composition were very similar between the natural community (t0) and after incubation (controls), with similar ASVs detected and relative abundances for the groups ‘mixotrophs’, ‘photoautotrophs’, and ‘trophy undetermined, including ‘unclassified Dinophyceae’, while changes did occur for the phagotrophs (Fig. 5). In fact, many phagotrophic classes were reduced or disappeared with incubation. In contrast, the class Bicoecea noticeably increased in all controls while other taxa (e.g. heterotrophic Dinophyceae, MAST, Telenomia, Spirotrichea, Litostomatea) were significantly reduced or absent from the samples after incubation (Fig. 5).

### Nutrient response of the protist community

(i) Diversity and composition

**Figure 6.**
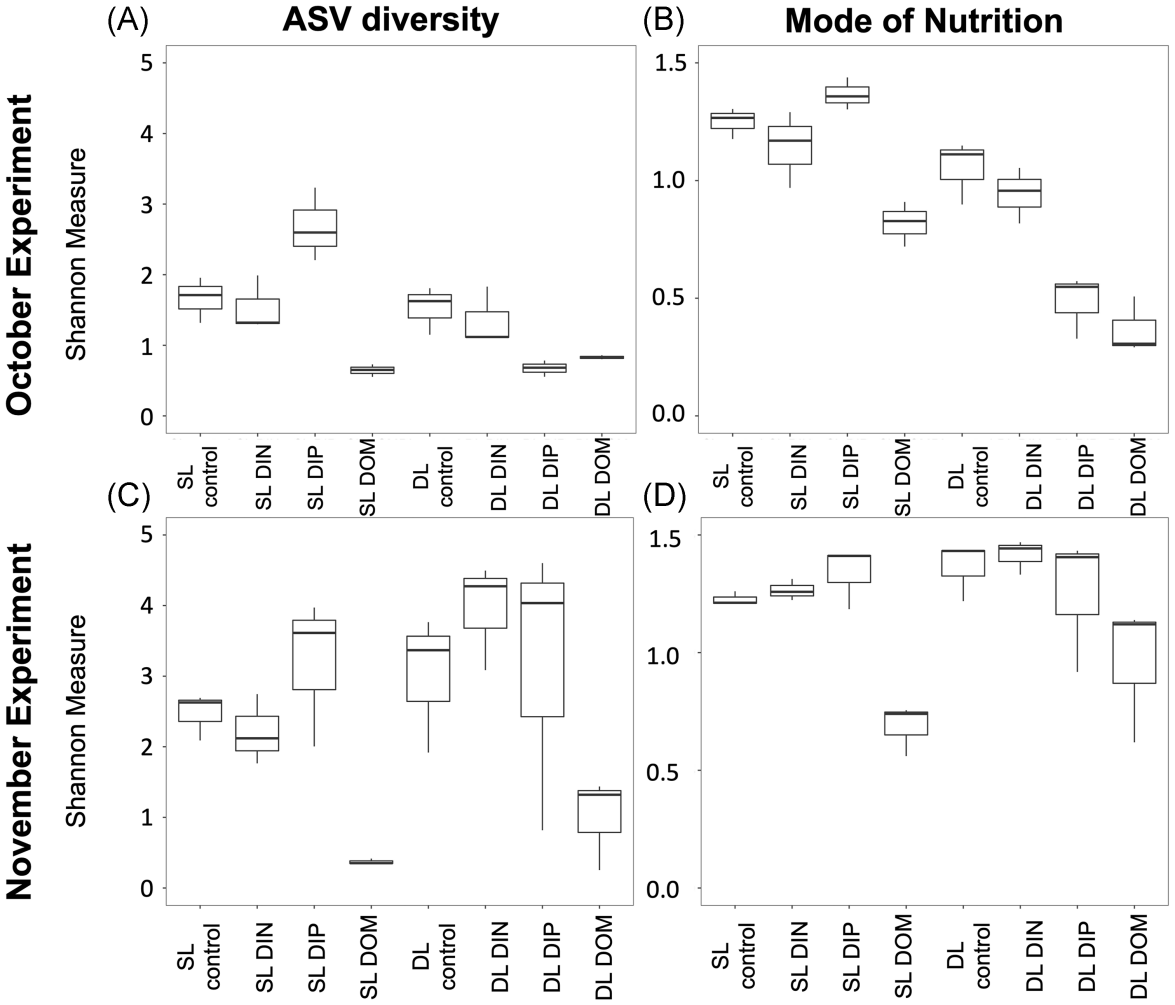
Comparison of Shannon measures of diversity between the different experimental treatments (controls; dissolved inorganic nitrogen, DIN; dissolved inorganic phosphorus, DIP; dissolved organic matter, DOM) by ASV diversity (**A, C**) and mode of nutrition (**B, D**) for October (**A, B**) and November (**C, D**) 2020 in both the surface layer (SL) and the deep layer (DL).

**Figure 7.**
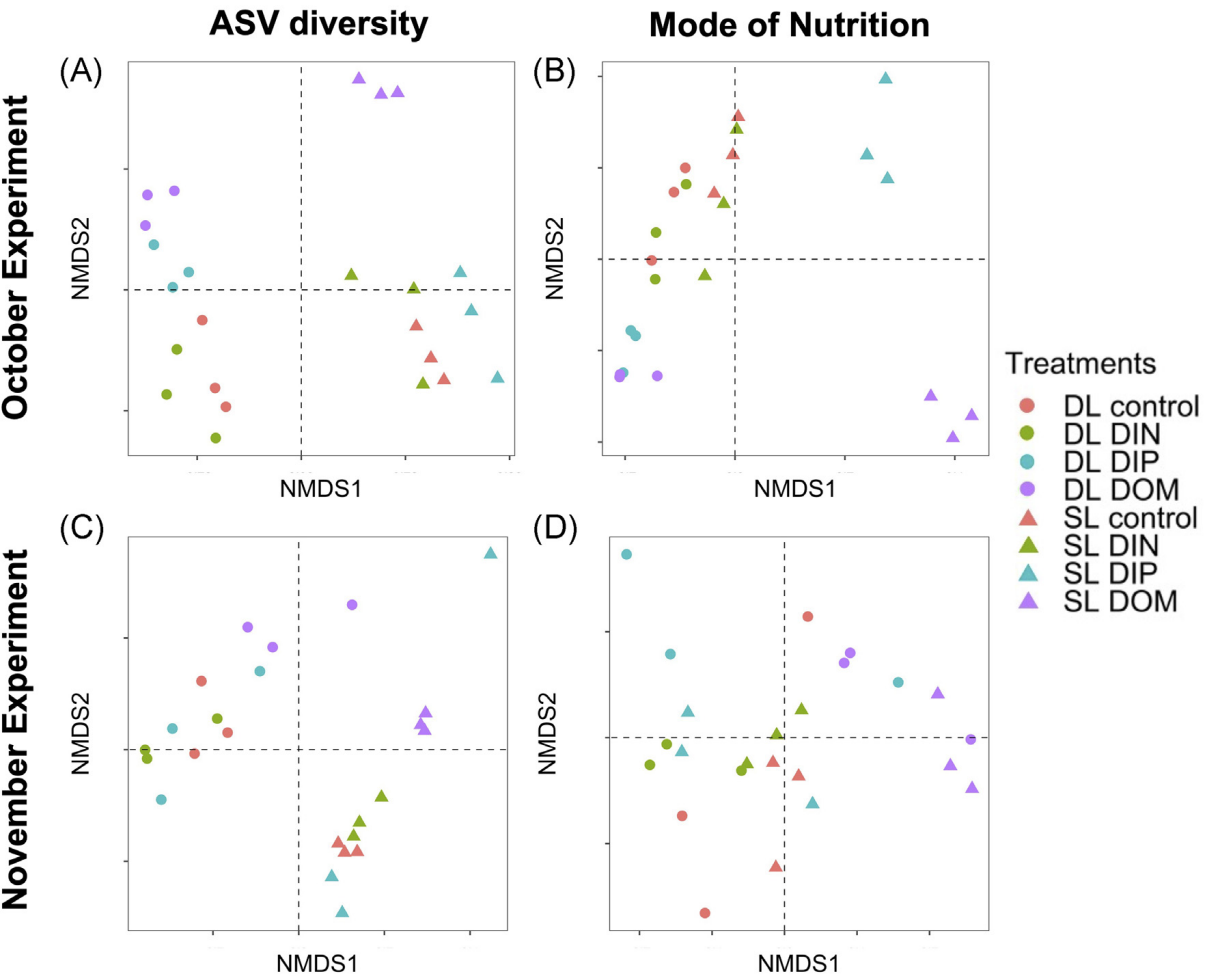
Non-metric multidimensional scaling (nMDS) ordinations of sample composition in the different experimental treatments (controls; dissolved inorganic nitrogen, DIN; dissolved inorganic phosphorus, DIP; dissolved organic matter, DOM) in terms of taxonomic composition (**A, C**) and nutritional mode (**B, D**) for October (**A, B**) and November (**C, D**) 2020 in both the surface layer (SL) and the deep layer (DL). Two-D stress indicated good fits (Kruskal, 1964) in all nMDS ordinations, with values less than 0.1 (**A**, 0.04; **B**, 0.02; **C**, 0.06; **D**, 0.01).

No changes in the community taxonomic and trophic diversities and compositions were identified following the addition of DIN. The analysis of the Shannon diversity indexes of each treatment group revealed relatively similar values between the controls from the surface (SL) and deep layers (DL) and their respective DIN treatments (Fig. 6), indicating communities of similar levels of diversity during both experiments. In addition, the analysis of the β-diversities, based on Bray-Curtis dissimilarities of the relative abundances of ASVs, agreed with the assessment of Shannon diversity. The nMDS ordinations displayed distinct clusters with noticeable differences between treatments and initial samples indicative of shifts in community composition in terms of taxonomy and trophic strategy (Fig. 7), however, DL controls DL DIN treatments as well as SL controls and SL DIN treatments clustered together in NMDS ordinations of the taxonomy (Fig. 7).

For both experiments the analysis of the homogeneity of multivariate dispersion (ANOVA *P*-values and average distances to geometric median) and associated pairwise comparisons suggested that the response of the community was a treatment effect rather than a dispersion effect (*P*-values > 0.05). In other words, the observations were not influenced by the difference in composition within groups of samples (treatments) but by differences in composition between treatments.

The addition of DIP triggered changes in the community taxonomic and trophic diversities and compositions. In October, the Shannon index of the community taxonomic diversity increased in the DIP treatment in the SL and decreased in the DL (Fig. 6A). Statistical tests (i.e. Kruskal-Wallis rank sum test and subsequent Dunn's test) identified a significant *P*-value (*P* < 0.05) for the comparison of the species richness between DL control and DL DIP only. Observations of the trophic diversity followed the same patterns, with an increase in Shannon diversity in the DIP treatment in the SL and a decrease again in the DL (Fig. 6B), however none of the comparisons between controls and treatments produced significant values (Dunn's tests *P*-values > 0.05). Furthermore, the analysis of the ASV composition displayed strong taxonomic differences between the SL and the DL. Analysis of the ASV composition revealed a distinct cluster for the treatment DIP (Fig. 7A) in the DL which was well separated from the control and DIN treatment clustered together (Fig. 7A). In the SL however, the control, DIN and DIP treatments were clustered together (Fig. 7A). The analysis of the trophic strategies identified distinct clusters for DIP treatments from both the SL and DL (Fig. 7B). The SIMPER analysis identified a significant *P*-value (< 0.05) in the group ‘trophy undetermined’ for the comparison DL control vs DL DIP.

In November, the response pattern of the taxonomic composition in the SL was similar to the one observed in October: the highest Shannon indexes corresponded to the DIP treatments again (Fig. 6C). However, the DIP treatment in the DL did not exhibit a decline in diversity in November as they did in October. Differences in ASV richness for November were not statistically significant (Kruskal- Wallis rank sum test; *P*-value > 0.05). Analysis of the trophic diversity revealed similar patterns to October (Fig. 6B), with high Shannon indexes in the SL for both DIN and DIP treatments and a slight decrease in DL for DIP (Fig. 6D). Differences in trophic diversity were not significant though (Kruskal- Wallis rank sum test; *P*-values > 0.05). The analysis of the ASV composition suggested strong differences in composition between the SL and DL but did not identify distinct clusters for the treatments DIN and DIP for either layer (Fig. 7C). Clusters were less identifiable for the trophic strategy with no clear differences between the layers (Fig. 7D). No significant differences were identified by the SIMPER analysis between the treatments and the control (*P*-values > 0.05).

Whilst changes driven by the addition of DIP were detected during both experiments, there were differences in the physiological responsiveness of the community between October and November. In fact, the ANOSIM analysis of the Bray-Curtis distance matrices revealed significant differences in treatments (*P*-value < 0.05) in microbial community composition and trophic group composition between treatments for both the experiments. Dissimilarity between treatments was, however, higher for October (October R values > November R values; Table 2). The PERMOVA analysis also pointed to significant differences in ASV composition between treatments (*P*-value < 0.05) both in terms of taxonomy and trophic strategy. In October, ∼ 84% of the dissimilarity in taxonomic composition and ∼ 97% for trophic composition could be both explained by treatment (Table 2). In November, the influence of the treatments on both the taxonomic and the trophic composition was reduced to ∼ 61% and ∼ 68%, respectively (Table 2). Pairwise comparisons using permutation MANOVAs on the distance matrices did not identify pairs of samples for which dissimilarity changes in taxonomic and trophic community composition were significant (*P*-values < 0.05).

**Table 2.** Bray–Curtis distance statistics.

Statistics	October Experiment		November Experiment	
Analysis	Taxa	Trophic groups	Taxa	Trophic groups
**ANOSIM**				
Significance	0.001	0.001	0.001	0.001
R	0.8935	0.818	0.668	0.432
**PERMANOVA**				
*P*-value	0.001	0.001	0.001	0.005
Treatments r^2^	0.841	0.966	0.605	0.681

The addition of DOM led to a loss of diversity and significant changes in community composition. During both experiments, the community response to DOM addition showed lower Shannon indexes in both layers compared to the controls for both the taxonomic and trophic diversity (Fig. 6). Statistical tests (i.e. Kruskal-Wallis rank sum and Dunn's tests) did not identify significant differences (*P* > 0.05) in taxonomy in either sampling month between controls and DOM treatments, although a significant difference (*P* < 0.05) was found for the comparison of trophic diversity between DL control and DL DOM in October. In November, no significant differences were identified between the controls and their respective DOM treatments (*P*-values > 0.05).

Furthermore, the analysis of the β-diversity for the species composition in October and November revealed distinct clusters from the controls for the DOM treatments at both depths (Fig. 7A, C). The analysis of trophic strategy also identified distinct clusters for the DOM treatments for both depths (Fig. 7B, D). The SIMPER analysis supported differences in composition between respective controls and DOM treatments of both layers in October for all the trophic groups (*P*-values < 0.05), with the exception of the mixotrophs (*P*-value > 0.05) in the SL while DL differences in composition were significant for mixotrophs and ‘trophy undetermined’ (*P*-values < 0.05). In November, the SIMPER analysis indicated that significant differences between the control and the DOM treatment in SL corresponded to the mixotrophs and ‘unclassified Dinophyceae’ (both *P*-values < 0.05), while no significant differences in trophic composition were identified in the DL between the treatment and control (*P*-values > 0.05).

To summarise, outputs of the examination of the Shannon and β-diversities as well as the taxonomic and trophic compositions were in good agreement with each other. Contrary to DIN treatments, the addition of DIP was responsible for significant changes in the community. However, the pattern in community response differed in the deep layers between October and November. The addition of DOM led to a loss of diversity and changes in community trophic composition during both experiments.

(ii) Changes in relative proportions of the eukaryotic taxa and response of the photosynthetic prokaryotes

**Figure 8.**
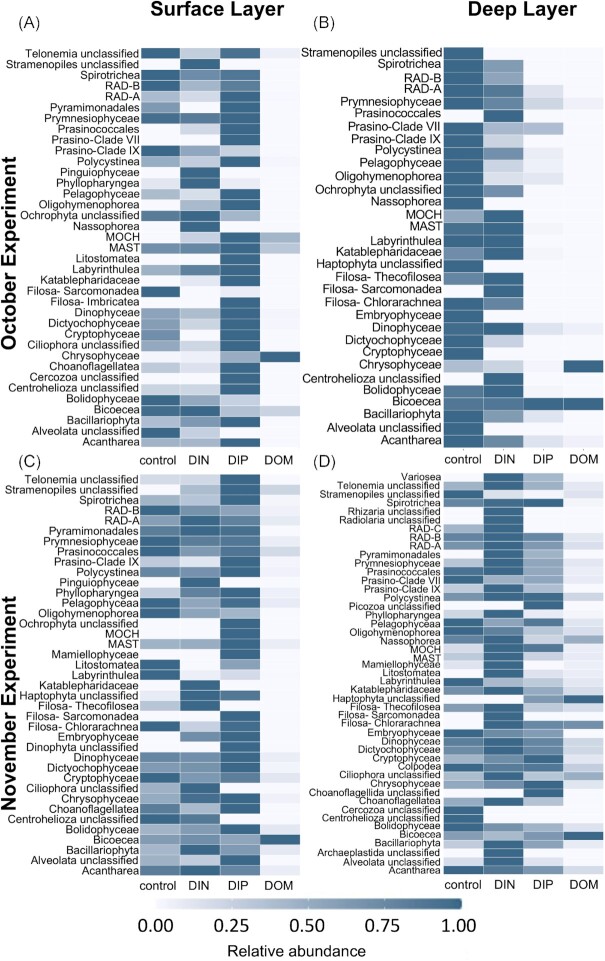
Comparison of the relative abundances of each taxonomic class between treatments (controls; dissolved inorganic nitrogen, DIN; dissolved inorganic phosphorus, DIP; dissolved organic matter, DOM) in communities sampled both at the surface layer (**A, C**) and deep layer (**B, D**) for the experiment of October (**A, B**) and the experiment of November (**C, D**) 2020. Saturated blue tiles corresponded to the treatments with the highest relative abundances of each class while white tiles corresponded to the treatment with the lowest relative abundance. Heatmaps showing the relative abundances for the individual replicates of the November experiment are available in Fig. S3.

The relative proportions of the eukaryotic taxonomic classes were similar between the controls and the DIN treatments (Figs 7 and 8). Examination of the relative abundances of each class between the controls and treatments was particularly consistent with the observation of both the Shannon diversity index and β-diversity. In October and November, the highest abundances of many classes, regardless of their trophic modes (e.g. Choanoflagellatea; Chrysophyceae; Dinophyceae; Mamiellophyceae [only detected in November]), were found in the DIP treatment in the SL (Fig. 8A, C). However, the addition of DIP triggered a noticeable decrease in most classes in the DL in October with the exception of Bicoceae (Fig. 8B), whereas the DIP treatment and control were similar in November (Fig. 8D). The addition of DIN triggered an increase in most classes in the DL community in November (Fig. 8D), in agreement with the observations of an increased Shannon diversity index (Fig. 6C). The only exceptions were Bicoceae, for which the relative proportions were the same in all treatments (control, DIN, DIP), Bolidophyceae, and the ‘trophy undetermined’ groups Centrohelioza, Cercozoa, Choanoflagellida, Haptophyta, Picozoa and Stramenopiles, all more abundant in the control (Fig. 8D). Finally, relative abundance of many classes decreased in the DOM treatments, except for Chrysophyceae in October (Fig. 8B), Bicoecea in the DL in October and in both layers in November (Fig. 8B, C, D), and Haptophyta with undetermined trophy in the DL in November (Fig. 8D), which were all more abundant in the DOM treatments compared to the controls.

**Figure 9.**
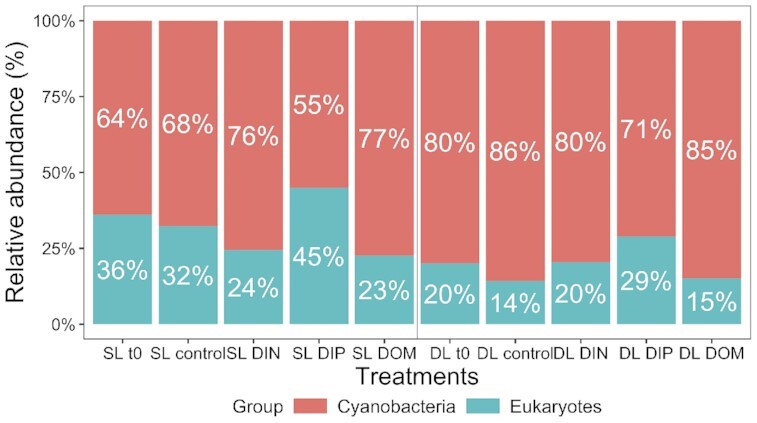
Relative abundances (%) of cyanobacteria, including *Synechococcus* and *Prochlorococcus*, and eukaryotes determined by flow cytometry (FL3/FSC) for each treatment (before incubation, t0; after incubation, controls; dissolved inorganic nitrogen, DIN; dissolved inorganic phosphorus, DIP; dissolved organic matter, DOM) in communities sampled both in the surface layer (SL) and deep layer (DL) in November 2020.

In November, relative abundances of cell counts of photosynthetic eukaryotic (photoautotrophs and mixotrophs) and prokaryotic (cyanobacteria including *Synechococcus* and *Prochlorococcus*) particles detected by flow cytometry (cell diameters < 10 $\mu $m) showed similar profiles between samples before and after incubation (t0s versus controls) in both the SL and DL (Fig. 9). After incubation, normalised counts of particles indicated that the highest abundances of photosynthetic eukaryotes were in the DIP treatments for both layers (Table 3, see also Fig. 9). When light was also a limiting factor (for DL), the addition of DIN triggered the growth of photosynthetic eukaryotes (Table 3), while photosynthetic prokaryote growth increased only following the addition of DIN to both layers (Table 3). In the DOM treatments, flow cytometry counts of photosynthetic particles after incubation, both prokaryotic and eukaryotic, showed a reduction in the SL while abundances were unchanged at depth (Table 3). Photosynthetic prokaryotes (cyanobacteria) were numerically dominant when compared to photosynthetic eukaryotes in all samples and with the highest relative proportions in the DOM treatment at the SL and in the DOM and control treatments at the DL (Fig. 9). The highest cyanobacteria abundances were found in the DIN treatments (Table 3).

(iii) Functional composition

**Table 3.** Normalised counts of photosynthetic prokaryotes (i.e. cyanobacteria including *Synechococcus* and *Prochlorococcus*) and photosynthetic eukaryotes determined by flow cytometry (FL3/FSC) in both the surface layer (SL) and deep layer (DL) for all treatments (i.e. controls; dissolved inorganic phosphorus, DIP; dissolved inorganic nitrogen, DIN; dissolved organic matter, DOM)

Treatments	Cyanobacteria	Eukaryotes
SL control	1.00	1.00
SL DIP	0.88	**1.50**
SL DIN	**1.28**	0.87
SL DOM	0.81	0.50
DL control	1.00	1.00
DL DIP	0.97	**2.37**
DL DIN	**1.31**	**2.02**
DL DOM	0.94	1.01

**Figure 10.**
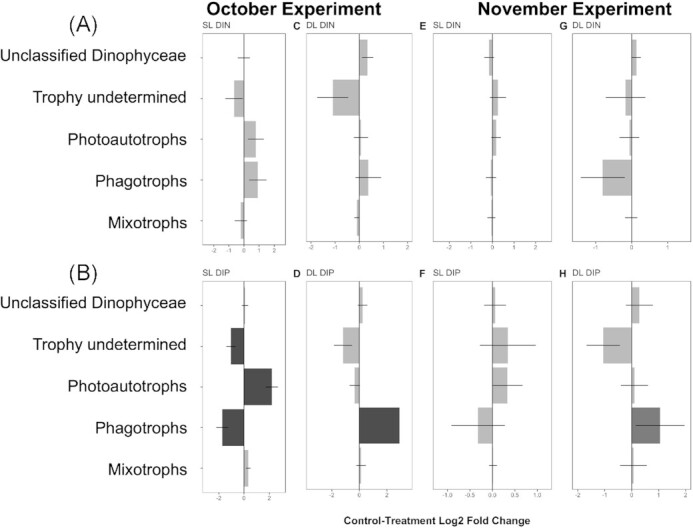
Magnitude of change in trophic groups between treatments (dissolved inorganic nitrogen, DIN; dissolved inorganic phosphorus, DIP) and controls in communities sampled both in the surface layer (SL) and deep layer (DL) for the month of October**(A)** and November**(B)**. Magnitude of change is expressed in log2 fold change, as estimated by the DESeq2 analysis. Dark bars represent groups for which the change was found to be statistically significant (*P*-value < 0.05, two-sided Wald test corrected with the Benjamini & Hochberg method). The significance of changes in phagotrophs in the treatment DIP at the DL in November (H) was not determined (*P*-value = NA) due to the presence of an extreme count outlier detected by Cook's distance. Horizontal lines show the standard error.

The DESeq2 analysis conducted on trophic groups (Fig. 10) revealed changes in trophic groups between treatments and controls, while a DESeq2 analysis on taxonomic groups revealed which taxa were likely responsible for these changes (Supplementary Table S7). Generally, changes were consistent between the experiments showing the same trends between treatments (Fig. 10). No significant changes (*P*-values > 0.05) in trophic group or taxonomic composition were observed for DIN treatments in both layers in October (Fig. 10A, C and Supplementary Table S7). In November, no significant differences in trophic composition were identified in the DIN treatments (Fig. 10E, G), however the DESeq2 analysis of the taxonomy identified a significant reduction in the phagotroph *Sagenista* in the SL (*P*-value < 0.05, Supplementary Table S7). DIP treatments did not show significant changes for taxonomy or trophic strategy in either layer in November (Fig. 10F, H), although the pattern in variability were very similar to the community response of the October experiment. In October, the DIP treatments triggered opposite effects on the SL and DL communities (Fig. 10B, D). The addition of DIP was responsible for a significant increase in relative abundance of photoautotrophs (Ochrophyta class Chrysophyceae but decreases in Bolidophyceae and Palmophyllophyceae) while ASVs with ‘undetermined trophy’ and phagotrophs decreased (Opalozoa) in the SL (Fig. 10B; Supplementary Table S7 for details of DESeq2 on the taxonomic classic). In the DL, the DIP treatment was responsible for an increase in phagotrophs (Fig. 10D; no taxa associated, Supplementary Table S7).

**Figure 11.**
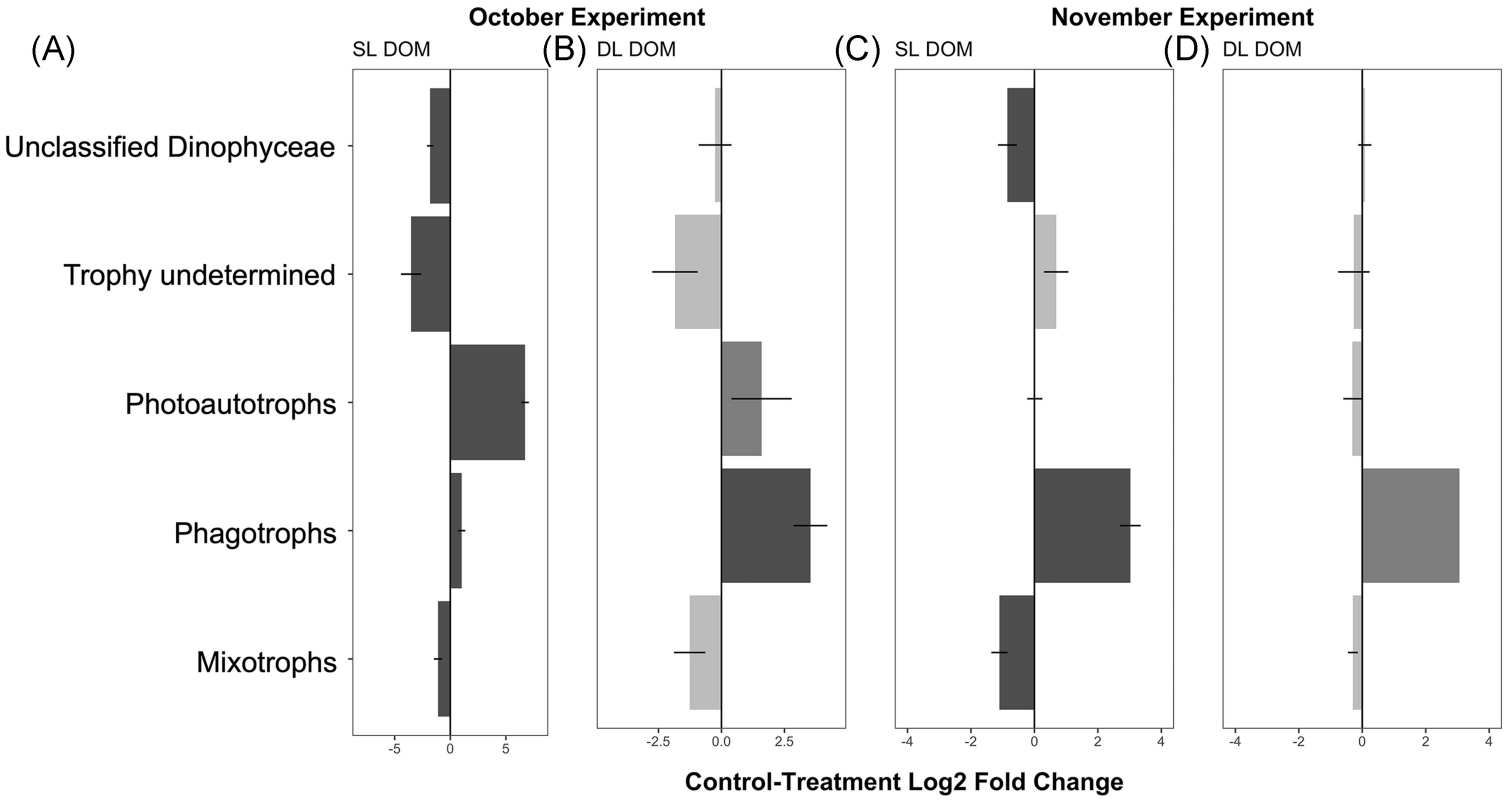
Magnitude of change in trophic groups between treatments dissolved organic matter (DOM) and controls for communities sampled in both the surface layer (SL) and deep layer (DL) in October **(A, B)** and November **(C, D)** 2020. Magnitude of change is expressed in log2 fold change, as estimated by the DESeq2 analysis. Dark bars represent groups for which the change was statistically significant (*P*-value < 0.05, two-sided Wald test corrected with the Benjamini & Hochberg method). The photoautotrophs change significance at the DL (**B**) in October 2020 as well as the phagotrophs change significance of at the DL (**D**) were not determined (*P*-value = NA) due to the presence of an extreme count outlier detected by Cook's distance. Horizontal lines show the standard error.

Differences in the relative proportions of trophic groups occurred between both experiments in the DOM treatments. The general trend showed an increase in relative abundances of phagotrophs in all samples (Fig. 11). In October, our analyses identified significant increases (*P*-values <0.05, Supplementary Table S7) in Bicoecea in the SL and Bicoecea, MAST and Oligohymenophoreae in the DL. In November, in the SL there was a significant increase in Bicoecea while Labyrinthulea decreased significantly and in the DL Choanoflagellatea decreased significantly (*P*-values <0.05, Supplementary Table S7). Photoautotrophs increased in both layers in October (Fig. 11A, B). DESeq2 analysis identified an increase in the class Chrysophyceae in the SL, while in the DL significant increases in Chrysophyceae and MOCH and significant reductions in Bolidophyceae, Prasino-Clade-IX and Pelagophyceae occurred (*P*-values < 0.05, Supplementary Table S7). The genus *Poterioochromonas* was the taxa associated with an increase in photoautotroph Chrysophyceae. While no significant changes in the photoautotrophs was detected in November through the DESeq2 functional analysis, the DESeq2 analysis of the taxonomy identified a significant increase in phototroph Dinophyceae (*P*-value < 0.05, Supplementary Table S7). Relative abundances of taxa with ‘undetermined trophy’ decreased significantly in October in the SL (Fig. 11A), with no associated taxonomic changes (*P*-values > 0.05, Supplementary Table S7). Mixotrophs were slightly reduced in all treatments although changes were significant only in the SL during both experiments (Fig. 11A, C), DESeq2 of the taxonomy identified significant decrease in the mixotrophs Prymnesiophyceae in all treatments in October only (*p-*values < 0.05, Supplementary Table S7). As for mixotrophs, ‘unclassified Dinophyceae’ were slightly reduced in most treatments although changes were significant only in the SL during both experiments (Fig. 11A, C), DESeq2 of the taxonomy did not identify significant differences (*p-*values > 0.05, Supplementary Table S7).

## Discussion

The nutrient enrichment assay coupled with 18S metabarcoding revealed the potential of natural microbial communities to rapidly shift in response to nutrient additions. Outputs highlighted the importance of phosphorus availability, largely understudied when compared to nitrogen, and its role in constraining microbial diversity. Dissolved organic matter addition led to a loss of diversity, benefiting a limited number of phagotrophic and mixotrophic taxa, although results were dependant on the pre-existing nutrient availability, in terms of total amount and ratios between the nutrients. The differential effect of each nutrient, and the dependency of the previous state of the ecosystem, should be then considered when modelling future conditions of the oceans in terms of changes in nutrient availability and flux in oligotrophic oceans, especially during stratified conditions.

### Vertical distribution of the natural plankton community is homogenous in the mixed layer

The taxonomic distribution of the eukaryote community agreed with several published studies in oligotrophic waters (e.g. see Detailed in Supplementary text 3). Broadly, our vertical profiles of the natural eukaryote community and hydrography were similar to those sampled in the fall at the BATS sites between 2016 and 2018 in the seasonal description of Blanco-Bercial et al. (2022). The photosynthetic picoplankton were numerically dominant in the euphotic zone, as is typical in oligotrophic regions (Mojica et al. 2015, Cotti-Rausch et al. 2016, Otero-Ferrer et al. 2018, Mena et al. 2019).

There was a homogenous community in the mixed layer, and differentiation of the protist functional and taxonomic distribution below the DCM (see Fig. 2 and Table S3). This pattern in differentiation of the protist community very likely followed the differentiation of the nutrient availability and light limitation gradients, both strengthening with depth. Ocean dynamics (e.g. summer stratification followed by fall mixing, nutrient availability and light irradiance) shaped the structure of the protist community expressed in the vertical partitioning of the community taxonomic and functional composition, as well as diversity (Blanco-Bercial et al. 2022). For instance, the paucity of nutrients favoured mixotrophic and phagotrophic protists in the surface ocean while decreasing irradiance along the depth gradient was likely the main factor responsible for the dominance of these taxa below the DCM. On the other hand, the highest proportion of phototrophs was found at the interface between the base of the euphotic layer and the top of the nutricline (see Fig. 1D) where light may not be limiting due to replete nutrients. This dynamic partitioning of the water column, with vertical niches depending on nutrient availability and hydrography, further supported the importance of understanding nutrient-driven changes in microbial community structure and physiology.

### Bottle effects

Overall, the observations of ‘bottle effects’ on the taxonomic richness as well as taxonomic and trophic compositions of the micro-eukaryote community (< 200 $\mu $m) supported the validity of the experimental design in including their natural diversity for the examination of the ecological interactions within the photosynthetic community (photoautotrophs and mixotrophs) and the interactions between the photosynthetic community and heterotrophic community (microzooplankton in this study), as well as the reproducibility of the experiments.

The ‘bottle effect’, defined in the literature as a reaction of the microbial community to batchwise incubation (Hammes et al. 2010), has been linked to changes in final cell concentrations (e.g. Bischofberger et al. 1990) and grazing/bacterivory behaviour (e.g. Marrase et al. 1992), a change in viability/activity (e.g. Jürgens et al. 2000), a change in cultivability (e.g. Ferguson et al. 1984), and/or a change in population composition (e.g. Agis et al. 2007). In our study, ‘bottle effects’ were seen as a change in the ASVs detected and ASV counts between the initial natural communities, and the communities after incubation, across most taxonomic and functional groups (Figs 4 and 5). A decrease in ASV diversity in the communities after incubation may allow some species to become more dominant in the DNA samples and rare ASVs within the reduced species number may be recognized greater in the outputs. However, the validity of the experimental design and its reproducibility were demonstrated by greater dissimilarities in ASVs composition between communities before and after incubations (> 70%) than among replicates (∼ 33%) (Tables S5 and S6). Furthermore, bottle effects did not affect the relative abundance of ASVs within the functional groups. The loss was likely a consequence of strong competition between taxa occurring in the incubation bottles, which seemed to favour the phagotrophic Bicoecea. The removal of larger taxa (> 200 $\mu $m) through pre-filtration likely relieved the smaller grazers (< 200 $\mu $m) from predation pressure and led to an increase in phagotrophs (Fig. 4). Surprisingly this change was not however associated with a decline in photoautotrophs or mixotrophs, their potential prey.

The unchanged relative abundances of photoautotrophs and mixotrophs, as well as an increase of relative abundances of photoautotrophs (surface layer during October), were both regardless of an increase in phagotrophs and likely implies that the photosynthetic community was capable of growing well within the incubation bottles, enough to support the grazing pressure. The phagotrophs present in the incubation bottles may not have been primarily feeding on the eukaryotic photosynthetic community but rather the prokaryotic community (both non-photosynthetic and photosynthetic).

### Dissolved inorganic P constrains taxonomic and functional diversity of small eukaryotes

While diversity indexes revealed a stronger response of the microbial community during the experiment in October, in both experiments the addition of nutrients triggered taxonomic and trophic changes (Figs 6 and 7). However, the differences in response between October and November, as well as in the surface layer versus deep layer, highlight the importance of the plankton community's prior exposure to nutrients.

Outputs from the nutrient enrichment assay indicate that P availability may play an important role in influencing the taxonomic and trophic diversity of micro-eukaryotic assemblages in oligotrophic oceans. Taxonomic differentiation of the micro-eukaryotic assemblages in the DIP treatment in the deep layer of October 2020 (Fig. 7A) occurred in parallel with a decrease in diversity (Fig. 6A). In the same way, the trophic differentiation of the eukaryotic assemblages in both the surface and the deep layers correlated with increases in diversity and significant changes in trophic group relative abundances. On the other hand, the DIN treatments did not result in any response from the eukaryotic community.

These results highlight the importance of the inorganic P in the nutrient-depleted surface layer during stratification in controlling the microbial community differentiation and in maintaining biodiversity. The importance of P in shifting the functional composition of pico-/nanoeukaryotic communities has already been reported for mixotrophs and strict heterotrophs (Wang et al. 2020), however in this prior study the percentages of photoautotrophs was not affected, explained under the assumption that photoautotrophs have the ability to produce non-phosphorus membrane lipids during P-limited growth (Van Mooy et al. 2009). While this is indeed a survival strategy found in several algal groups, we found contrasting results in our dataset, which instead support the existence of a relationship between P and photoautotroph abundance. Similar observations were reported in a DNA-metabarcoding study of the mixoplankton community in the Belgian coastal zone, where inorganic P availability was the most important factor driving diversity and species richness of mixotrophs, autotrophs and heterotrophs, particularly in fall and winter (Lapeyra Martin et al. 2022). In short, N may be limiting in terms of physiology, biomass production, and cell abundance, particularly for photosynthetic prokaryotes; P, however, seems to be influencing the taxonomic diversity of the plankton community, moderating the growth of photosynthetic eukaryotes, and might play a role in shifting the functionality of micro-eukaryote communities. In fact, the cyanobacterial community was affected only by the addition of inorganic N while the photosynthetic eukaryotes primarily responded to the addition of P (Table 3).

These variations in response between the communities suggest an important trade-off that may influence seasonal patterns in biodiversity, abundance and ecological function in regions with summer stratification is followed by fall mixing. Additionally, if nutrient input to surface waters is reduced, due to the low atmospheric input of P into the open ocean contrary to N (Broecker and Peng 1982), P might become even more limiting. Therefore, it is not unreasonable to hypothesize that the biodiversity of microbial eukaryote communities will decrease alongside decreasing P availability and, ultimately, lead to lower primary production (e.g. Krom et al. 2010). Biodiversity promotes primary productivity and its temporal stability (Oehri et al. 2017). While open ocean data have shown an increase in species dominance across a gradient of nutrient availability and highlighted the way marine phytoplankton sustain world food stocks through species selection in communities containing a high taxonomic diversity, Cermeño et al. (2016) suggested that the preservation of photosynthetic plankton species richness is essential to sustain ocean primary productivity since it guarantees the occurrence of highly productive species.

### Taxonomic and functional diversities are coupled

Our study highlighted the importance of nutrients in modulating functional diversity. Studies have already investigated trophic strategies of protist communities based on 18S metabarcoding data (e.g. Charvet et al. 2014, de Vargas et al. 2015, Wang et al. 2020). Environmental genomic and transcriptomic analyses of marine prokaryotes have shown that diverse taxonomic communities could express similar functional roles through shared metabolic pathways (Louca et al. 2016, Coles et al. 2017, Haggerty and Dinsdale 2017), suggesting that functional and taxonomic diversity are decoupled and functional diversity is constrained by environmental conditions while the taxonomic diversity is driven by biotic interactions (Louca 2017, Louca and Doebeli 2017). In our study, however, environmental parameters, such as P availability, drove both the taxonomic and trophic diversities of the micro-eukaryotic community. Examination of community diversity, differentiation and composition (Figs 6, 7, 10 and 11) all pointed to taxonomic and functional compositions being linked. Further, the contrast seen between protists and prokaryotes could be explained by the level of expression of their functionality.

The functional roles of prokaryotes in their environment are explained at the molecular and metabolic level, for instance by enzyme development in parallel with biogeochemical cycles (Falkowski et al. 2008). In contrast, eukaryotes express their functional role at the cellular level, through behaviour, specialized morphology, adaptations and ecological strategies (Massana and Logares 2013) that separately evolved across multiple micro-eukaryote taxa. Therefore, protist functional diversity could be linked to both their taxonomic diversity and the environment they experience. Our results concur with Ramond et al. (2019) who found a tight coupling between taxonomic and functional diversity in marine planktonic protists in the Atlantic Ocean. We suggest that the nutrient status of the environment (i.e. eutrophic versus oligotrophic) in which a community exists (or its nutrient history), influences how strongly taxonomy and trophy are coupled or decoupled. Furthermore, the coupling between taxonomy and functionality are stronger in the oligotrophic ocean where photosynthetic and heterotrophic protists have differentiated further to adapt to the extreme environmental conditions of chronic nutrient-depletion and low primary productivity. As oligotrophic areas are expanding, determining the mechanisms underlying shifts in the functionality of microbial communities and ecosystems driven by environmental parameters and/or biotic interactions will help make predictions of the microbial community structure, biodiversity, ecosystem productivity, and carbon export.

### The use of dissolved organic matter by protists

The response of our prokaryotic communities to the introduction of DOM (i.e. an increased availability of dissolved organic carbon), agrees with previous observations of increases in bacterioplankton productivity (e.g. Cole et al. 1988, Baines and Pace 1991), leading to a higher abundance of heterotrophic (bacterivorous) eukaryotes (Lonsdale et al. 2006). In our study, this was indicated by an increased relative abundance of phagotrophs in most of our DOM treatments, especially the Bicoecea (Fig. 11). This is consistent with the fact that small heterotrophic protists are the primary consumers of prokaryotic and eukaryotic biomass (Burkill et al. 1993, Landry et al. 1997, Boenigk and Arndt 2002, Sherr and Sherr 2002), as well as promoting organic matter remineralization (Boenigk and Arndt 2002). The importance of dissolved organic carbon for nano- and bacterioplankton distribution has already been reported for communities of the Sargasso Sea (Burney et al. 1981). In our case, the addition of DOM led to a strong reduction in biodiversity and to the dominance of a limited number of specific heterotrophic taxa. However, our dataset does not include data on heterotrophic prokaryote growth or diversity and includes little data on photosynthetic prokaryotes that might have played important roles (e.g. as competitors for resources or as nutrient resources themselves). While it is possible that the ingestion of DOM during the nutrient enrichment assay triggered growth of the heterotrophic groups, an alternative explanation could be an increase of heterotrophic bacteria prey.

Aside from the known obligate heterotrophs, there were also groups typically classified as photoautotrophs that had higher relative abundances in the DOM treatments. One such group that appeared to benefit from the DOM treatment were the Chrysophcyeae, whose members include a wide spectrum of nutritional capabilities from obligate phototrophy, through varying levels of mixotrophy to osmotrophy and phagotrophy (Olrik 1998). Specifically, the genus *Poterioochromonas* increased in the DOM treatments for both experiments (Table S7). This genus is composed of mixotrophic flagellates, capable of both photosynthesis and phagotrophy, who are recognized as playing an important role in grazing bacteria and regenerating nutrients (Sanders et al. 1990, Zhang and Watanabe 2001). Mixotrophic Chrysophyceae and obligately heterotrophic Bicoecea are well adapted to feeding through particle-associated life-styles (Jeuck and Arndt 2013). These taxa often dominate microbial eukaryote assemblages at depths below the euphotic zone (> 200 meters) and are associated with low heterotrophic diversity (e.g. Schnetzer et al. 2011, Giner et al. 2020, Obiol et al. 2021), especially in labile carbon-limited deep ocean communities that rely almost exclusively on particulate organic matter flux from the surface ocean (Nagata et al. 2010).

Calcifying haptophytes (coccolithophores), another one of the groups that benefited from DOM addition (Table S7), have been demonstrated to use osmotrophy to provide nutritional flexibility and as a survival strategy to maintain their metabolism in light-limited conditions (Godrijan et al. 2020). In addition to their ability for osmotrophy, haptophytes can be major bacterivores in marine systems (Frias-Lopez et al. 2009, Unrein et al. 2014) and the mixotrophic genera of this group feed on a diverse spectrum of prey including both bacteria and other protists (Hansen and Hjorth 2002, Tillmann 2003).

Together, these results highlight the ability of Bicoecea, Chrysophyceae and Haptophyta to thrive on DOM and to exert high grazing pressure on prokaryotes and other small eukaryotes (Boenigk and Arndt 2002), at least in the absence of larger predators. In fact, it is likely that in the presence of larger (>200 µm) zooplankton the success of these taxa may have been balanced out, potentially allowing for the co-existence of other photosynthetic and heterotrophic taxa that were not detected in our study (Thingstad 2000, Winter et al. 2010).

## Conclusions

Our results demonstrated the importance of testing the effects of nutrient availability at the community-level, the results of which can significantly differ from laboratory observations and generate different mechanistic hypotheses. Inorganic P was identified as an important controller of biodiversity, while DOM addition reduced diversity in favour of heterotrophic assemblages.

We identified one homogeneous plankton community within the mixed layer in terms of taxonomic and functional composition, as opposed to vertically distinct communities found at each depth sampled below the DCM. Differentiation below the DCM increased along the depth gradient, highlighting strong changes in resource availability with depth. Critically, this pre-existing nutrient landscape had a significant effect on the results of experimental nutrient addition. As oligotrophic areas expand and vertical stratification strengthens, these patterns will become more important in driving the spatial and temporal dynamics of plankton communities in oligotrophic waters. Future studies need to consider water column structure when approaching similar experiments or modelling future responses of plankton communities.

Our findings suggest the need for further work to examine prokaryotic community (both autotrophic and heterotrophic) responses alongside small eukaryotic community dynamics. Future efforts that focus on linking changes in metabolism and physiology with changes in taxonomic and functional diversity are needed to improve our predictions of future plankton community structure, productivity, and biogeochemical roles.

## Supplementary Material

xtac029_Supplemental_File
