# Supplementary material for: Organic and inorganic nutrients modulate taxonomic diversity and trophic strategies of small eukaryotes in oligotrophic oceans
Source: FEMS Microbes. 2022 Dec 7;4:xtac029. doi: 10.1093/femsmc/xtac029 (PMC10117809; doi:10.1093/femsmc/xtac029)
Supplement: xtac029_Supplemental_File [file xtac029_supplemental_file.pdf]

**Manuscript ID: FEMSMC-2022-022 / Major revisions**

**Associate Editor's Comments to Authors:**
Associate Editor:
Thank you for your patience during the review process. Your manuscript has now been seen by three reviewers. As you will see, they all appreciate the manuscript, but two reviewers provide a range of comments and suggestions for improvement. Please address these carefully in your revision.

**Comment:** We would like to thank all three reviewers and the associate editor for their consideration of our manuscript and valuable recommendations on how to improve the article. We have worked through the specific comments of each reviewer and made corrections accordingly, below are our responses and details for the corrections made.

**Reviewer 1** (pages 2-14)– In addition to minor revisions, the manuscript now includes revised figures and legends, as well as a new supplementary figure (Fig. S3) and tables (Tables S5 and S6) to address the reviewer's comments on the bottle effects.

**Reviewer 2** (pages 15-19)– In addition to minor revisions, the introduction was shortened and re-structured as recommended by the reviewer and the list of literature was updated (e.g., Lapeyra Martin *et al*., 2022; Leles *et al*., 2019; Schneider *et al*., 2020). We also address in detail the reviewer's comment on rarefaction.

**Reviewer 3** (pages 20-31)– In addition to minor revisions, we present new figures (Figs. 2, 5, S1 and S2) and the associated text in the revised manuscript following the reviewer's recommendations. We also provide a detailed response to the reviewer's comment on the interpretation of the data in Figure 5.

Details of how we addressed each specific comment is listed below. We have indicated our corrections in blue text and the reference to line numbers (in MS Word as 'All markup' mode in 'Review') refer to the **new** version of the manuscript. We hope this fully satisfies the reviewers' comments and allows the paper to be considered further for publication.

**Reviewers' Comments to Authors:**
**<u>Reviewer 1:</u>**
Comments to the Author
A thorough study of protist communities in the Oligotrophic Sargasso Sea at different depths, and an assessment of the effects of N- and P- addition by means of batch experiments under natural conditions.
**Comment:** We would like to thank Reviewer 1 for their review of our manuscript and valuable recommendations on how to improve the paper.

Introduction
to the point and well-explained. aims clearly stated.

Mention in the Introduction already that the 18S V4 region is used. This is a commonly used region for metabarcoding but it is good to mention this as other or larger regions are beginning to be used nowadays.

**Response:** We thank Reviewer 1 for their suggestion. Mention of the use of the 18S V4 region in the introduction can be found at Lns 180-183: *'The small ribosomal subunit 18S gene and the region V4 are commonly used in metabarcoding studies, and can be used to determine the community diversity and species dynamics of aquatic eukaryotic protists (Martin et al., 2022).'*

The following reference was added:
Martin, J. L., Santi, I., Pitta, P. John, U. and Gypens, N. (2022) Towards quantitative metabarcoding of eukaryotic plankton: an approach to improve 18S rRNA gene copy number bias. MBMG, 6, e85794.

Methods
The authors hit the right balance providing essential information in the body of the text and putting the detailed information in Supplemental material. (write -20°C as -20 °C).

**Response:** This is now corrected for all temperatures in the manuscript.

PR2 database, maybe mention which version.

**Response:** We have now added the version information for the PR2 database. Lns 333-335: '*Taxonomic assignment was done using the PR2 database (version 4.14.0; Guillou et al., 2013), a database suited for protists.*'

Lines 264, 339, 359 etc. Dinophycean ASVs or reads make up a large proportion of the data. This is no surprise because specimens of this taxon tend to have far higher copy numbers of 18S rDNA in their haploid genomes than other lineages, creating a major skew in ASV and read abundances towards Dinoflagellates

**Response**: We thank Reviewer 1 for pointing this out, we now mention this in the methods section Lns 365-369: '*Importantly, deviation in the number of gene copies across plankton taxa (Not et al., 2009) can significantly affect the proportion of reads per taxa in complex microbial assemblages and lead to misinterpretation of relative abundances (Santi et al., 2021). This is particularly true in the case of Dinophyceae which tend to have a higher copy number of 18S rDNA than most lineages (Martin et al., 2022).*'

The following references were added:
Not, F., del Campo, J., Balagué, V., de Vargas, C. and Massana, R. (2009) New Insights into the Diversity of Marine Picoeukaryotes. PLoS ONE, 4, e7143.

Santi, I., Kasapidis, P., Karakassis, I. and Pitta, P. (2021) A comparison of DNA metabarcoding and microscopy methodologies for the study of aquatic microbial eukaryotes. Diversity, 13, 180.

Results

Line 312-317: Peaks of fluorescence … cyanobacteria .. whereas photosynthetic eukaryotes … 80 m in November. May I suggest to provide here in the text also info on where those cyanobacteria maxima were found to allow immediate comparison.

**Response:** We thank Reviewer 1 for their suggestion and we now provide this information in the following paragraph, Lns 454-457: '*Peaks of fluorescence and Chl-a concentration corresponded to the highest abundance of cyanobacteria found in the depth profiles, with a cyanobacteria maxima at ~ 90 m, while small photosynthetic eukaryotes (< 5 µm) were more abundant at ~ 80 m in November (Fig. 1C, D).*'

Lines 329-333: Apparently, it is not the numbers of ASV that changed but the ASVs themselves. If you talk about the numbers then I would expect info like 40-ish ASVs in the surface up to 650-ish in the DCM.

**Response:** The sentence has been modified, Lns 494-495: '*The ASVs detected in the samples also changed strongly across this vertical gradient.*'

Lines 336-351 I suggest stressing here that we talk here about amplicon reads of Dinophyceae rather than Dinophyceae. This is an issue because different plankton organismal groups exhibit radically different numbers of 18S copies per haploid genome. Stressing upfront that the mentioned abundances are based on amplicon reads will deal with that potential critique.

**Response:** Following Reviewer 1's suggestion, we now mention that the abundances are based on amplicon reads Lns 586-588: '*Relative abundances based on the contribution of amplicon reads of ASVs to total community amplicon reads, revealed that: […]*'. See also, Lns 506-508: '*Relative abundances based on the contribution of amplicon reads of trophic groups to total community amplicon reads, revealed that: […]*'.

We have also added the following sentence to the methods section, Lns 365-369: '*Importantly, deviation in the number of gene copies across plankton taxa (Not et al., 2009) can significantly affect the proportion of reads per taxa in complex microbial assemblages and lead to misinterpretation of relative abundances (Santi et al., 2021). This is particularly true in the case of Dinophyceae which tend to have a higher copy number of 18S rDNA than most lineages (Martin et al., 2022).*'

Lines 371-380 About the bottle-effect; community diversity is expected to diminish in a chaotic and randomised way, especially in small volumes of just a few L. It is therefore important that there are multiple replicates and that the extent of that differentiation is shown to answer the question what is reproducible trend and what is chaos, Just stating that the numbers of taxa (you probably mean ASVs) go down similarly with incubation time within the same treatment may not be good enough, because ASV composition is compared among treatments and far-reaching conclusions are drawn. This means that differences in composition need to be far less within replicates. Instead I read that "Specifically, a ~78% loss in taxa (=ASV) richness between t0 and controls in October and a ~59% loss in November" is a whopping lot. However, I am not an expert on statistics and the authors may have addressed this in their tests.
Anyway, answering this issue satisfactorily has consequences for the evaluation of the trends seen in the experiments

**Response:** We thank Reviewer 1 for making this important point, and apologise for not highlighting clearly that we do already address this issue. **To clarify the point** we now stipulate that while the number of ASVs decreased significantly between samples at t0 and after incubation (controls), *β*-

diversity showed that differences in community composition were far lower between replicate treatments (*n*= 3) than between t0s and controls. Lns 608-625: '*Examination of the number of ASVs in the samples indicated a loss of diversity in the samples after incubation (i.e., t0 versus controls). Specifically, a ~ 78 % loss in ASV richness between t0 and controls in October and a ~ 59 % loss in November. However, Bray-Curtis dissimilarity showed that the communities were compositionally similar between replicates (n = 3), with dissimilarities only around ~ 33 % (see Supplementary material; Figure S3, Table S5 for the month of October 2020, and Table S6 for the month of November 2020), while communities were compositionally different between t0s and controls with dissimilarities around ~ 81 % (Supplementary material, Tables S5 and S6).*'

**Table S5.** Bray-Curtis dissimilarity matrix of the bottle effects comparison between samples before incubation (t0) and after incubation (control) in both the surface layer (SL) and the deep layer (DL) for the month of October 2020. Grey boxes represent comparisons between replicates and blue boxes represent comparisons between t0s versus controls.

| October | | DL t0 | | | DL control | | | SL t0 | | | SL control | | |
|---|---|---|---|---|---|---|---|---|---|---|---|---|---|
| | | A | B | C | A | B | C | A | B | C | A | B | C |
| DL t0 | A | 0.00 | | | | | | | | | | | |
| | B | 0.26 | 0.00 | | | | | | | | | | |
| | C | 0.27 | 0.29 | 0.00 | | | | | | | | | |
| DL control | A | 0.86 | 0.86 | 0.85 | 0.00 | | | | | | | | |
| | B | 0.89 | 0.89 | 0.89 | 0.31 | 0.00 | | | | | | | |
| | C | 0.85 | 0.85 | 0.85 | 0.32 | 0.35 | 0.00 | | | | | | |
| SL t0 | A | 0.70 | 0.71 | 0.70 | 0.88 | 0.92 | 0.88 | 0.00 | | | | | |
| | B | 0.70 | 0.71 | 0.70 | 0.89 | 0.92 | 0.87 | 0.25 | 0.00 | | | | |
| | C | 0.70 | 0.71 | 0.70 | 0.88 | 0.92 | 0.88 | 0.27 | 0.26 | 0.00 | | | |
| SL control | A | 0.93 | 0.93 | 0.93 | 0.71 | 0.71 | 0.68 | 0.82 | 0.82 | 0.83 | 0.00 | | |
| | B | 0.93 | 0.93 | 0.93 | 0.66 | 0.67 | 0.65 | 0.85 | 0.84 | 0.84 | 0.31 | 0.00 | |
| | C | 0.90 | 0.91 | 0.90 | 0.66 | 0.64 | 0.66 | 0.75 | 0.78 | 0.78 | 0.38 | 0.38 | 0.00 |

**Table S6.** Bray-Curtis dissimilarity matrix of the bottle effects comparison between samples before incubation (t0) and after incubation (control) in both the surface layer (SL) and the deep layer (DL) for the month of November 2020. Grey boxes represent comparisons between replicates and blue boxes represent comparisons between t0s versus controls.

| November | | SL t0 | DL t0 | DL control | | | SL control | | |
|---|---|---|---|---|---|---|---|---|---|
| | | A | A | A | B | C | A | B | C |
| SL t0 | A | 0.00 | 0.71 | | | | | | |
| DL t0 | A | 0.71 | 0.00 | | | | | | |
| DL control | A | 0.83 | 0.79 | 0.00 | | | | | |
| | B | 0.78 | 0.72 | 0.48 | 0.00 | | | | |
| | C | 0.81 | 0.78 | 0.50 | 0.40 | 0.00 | | | |
| SL control | A | 0.73 | 0.90 | 0.66 | 0.64 | 0.58 | 0.00 | | |
| | B | 0.74 | 0.90 | 0.67 | 0.64 | 0.62 | 0.36 | 0.00 | |
| | C | 0.70 | 0.88 | 0.67 | 0.62 | 0.56 | 0.33 | 0.37 | 0.00 |

Discussion

A considerable section in the discussion is devoted to the bottle effect, and justly so.. There are two issues here that need to be addressed. One: in how far are the results of experiments due to bottle effects; and two: are the differences in taxonomic composition of ASVs among replicates far lower than the differences among experimental conditions. The first question is discussed, it is not crystal clear to me if the second has been addressed.

**Response:** We thank Reviewer 1 for again highlighting this point and refer to our last response (above) as well as discussing this, Lns 1090-1093: '[…] *validity of the experimental design and its reproducibility were demonstrated by greater dissimilarities in ASVs composition between communities before and after incubations (> 70%) than among replicates (~ 33%) (Supplementary material, Tables S5 and S6).*'

The authors write in lines 630-631 "In our case, bottle effects did not affect the relative abundance of species richness within most functional groups." I wonder what "relative abundance of species richness" means.

**Response:** For clarity, we have changed this sentence, Lns 1093-1094 to: '*Furthermore, bottle effects did not affect the relative abundance of ASVs within the functional groups*.'

Another reason for the bottle effect may be that the community at t0 is not the actual community as in the field. The zooplankton has been removed. Given this issue, the discussion is well structured, addressing a small set of major topics, presenting for each what is known from literature, and reflecting own findings against what is known. I did not find any over-interpretation of data.

**Response:** We thank the reviewer for this comment and recognising our wish not to over-interpret experimental field work based on community incubations, as well as our focus on relative differences between treatment replicates and different treatments to come to our conclusions.

An issue is ASV diversity versus species diversity. Especially with a decrease in ASV diversity in the experimental samples over time, some species may become more dominant in the DNA samples. This means that more of the rarer ASVs within those species will show up.

**Response:** We have corrected 'species diversity' to 'ASV diversity' in the relevant sentences to clarify this issue. We also added the following comment to the discussion to acknowledge it (Lns 1087-1090); '*A decrease in ASV diversity in the communities after incubation may allow some species to become more dominant in the DNA samples and rare ASVs within the reduced species number may be recognized greater in the outputs.*'

Line 745 Who = That

**Response:** Corrected, Lns 1238-1244: '*However, our dataset does not include data on heterotrophic prokaryote growth or diversity and includes little data on photosynthetic prokaryotes that might have played important roles (e.g., as competitors for resources or as nutrient resources themselves).*'

Literature is well covered

Figures are generally clear. In the one on page 37 the items of trophic composition are unreadable

**Response:** We thank Reviewer 1 for pointing this out. We have modified the featured image and made the items of trophic composition bigger, see below.

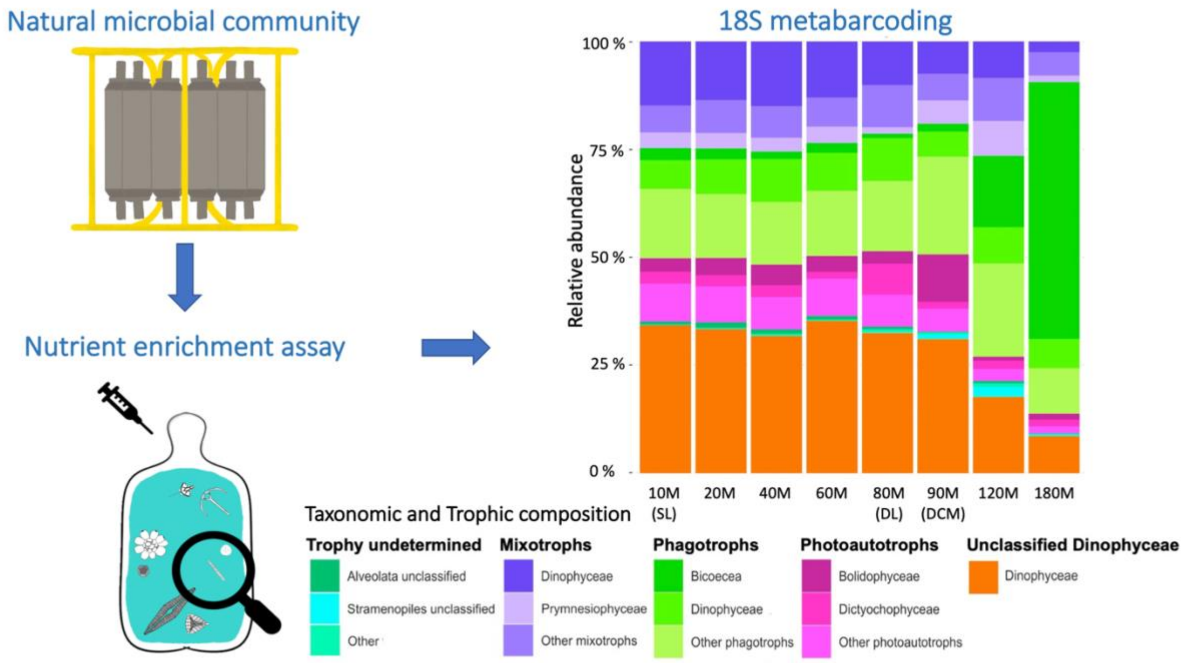


Fig. 1. Consider adding colour legend inset instead of writing colours in figure legend. There is space.

**Response:** Following Reviewer 1's suggestion, a colour legend has now been added to Figure 1, see below.

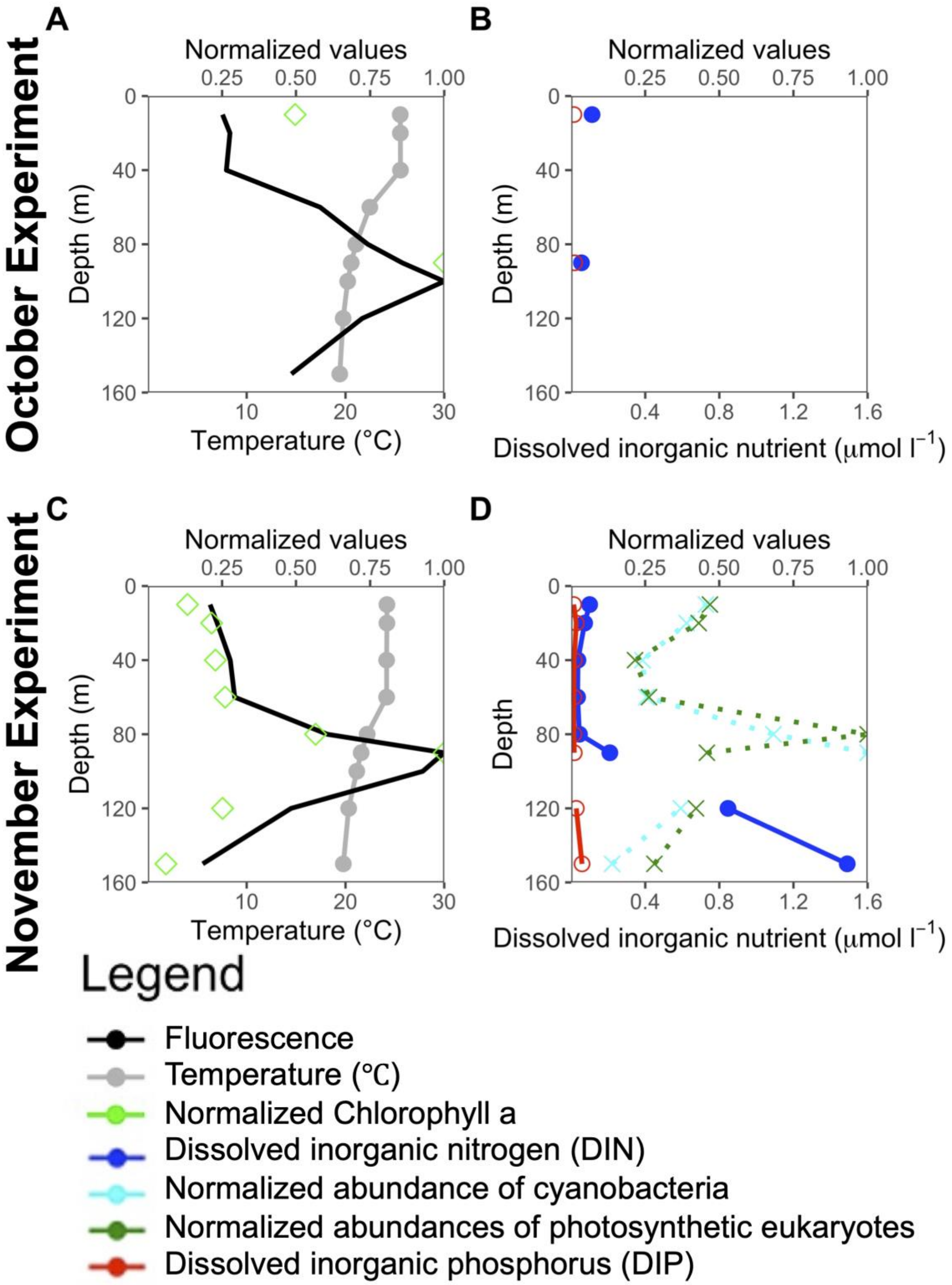


**Figure 1.** Vertical profiles of the physico-chemical and biological parameters at Hydrostation 'S' during sampling in October (A, B) and November (C, D). Temperature (grey dots) and fluorescence (solid black line) data correspond to *in situ* measurements recorded with a CTD. Normalized Chl-*a* (green diamonds), dissolved inorganic nitrogen (blue dots) and dissolved organic phosphorus (red dots), and normalized abundances of cyanobacteria (cross shape with dotted cyan line) and photosynthetic eukaryotes (cross shape with dotted green line) detected by flow cytometry (in November only), were all computed from samples collected with a rosette and processed in the laboratory.

Fig. 2A, though visually arresting, it is not very clear which block corresponds to which in the legend. The green one is probably Dinophyceae but that is not inherently clear.

**Response:** Following Reviewers 1 and 3's remarks, we have simplified Figure 2 and now provide details of the taxonomic composition (i.e., classes relative abundances of main phyla for each sample depth) in the **Supplementary material** (Figure S1).

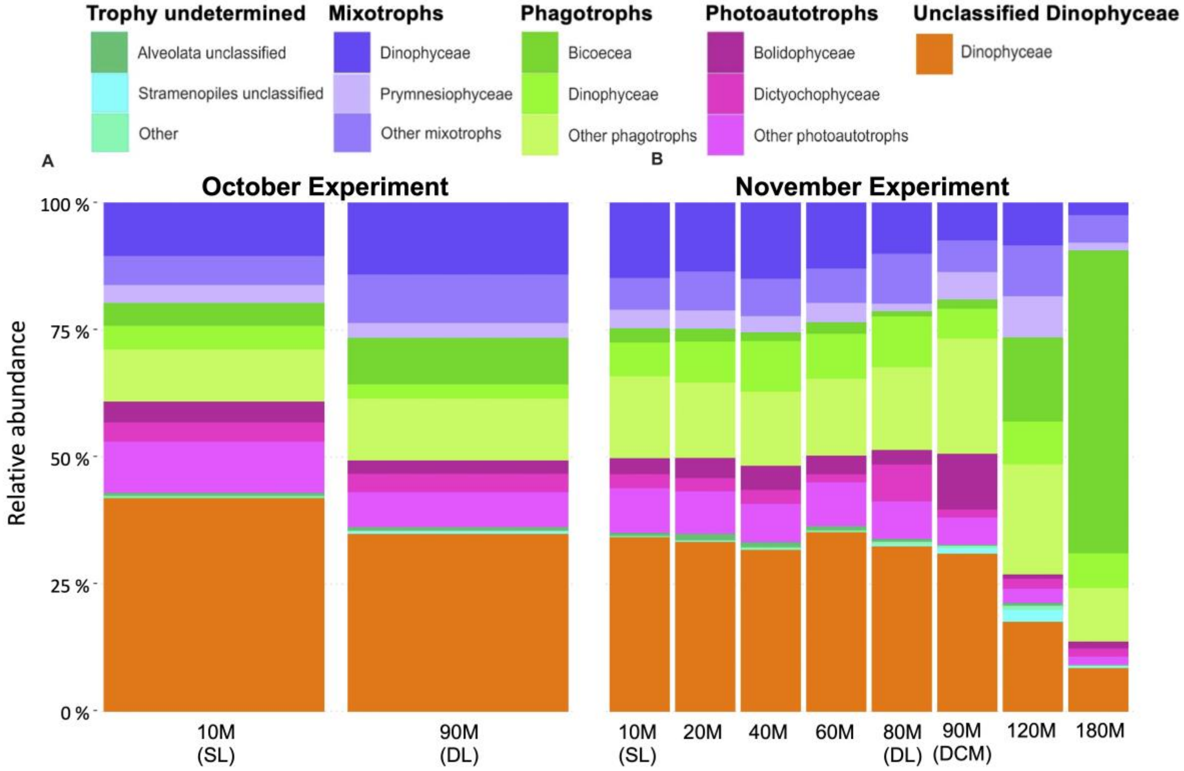


**Figure 3.** Taxonomic and functional composition (relative abundance, %) of the eukaryotic communities (excluding parasites and saprotrophs) in samples collected from different depths in the water column during October (A) and November (B). The two most abundant taxonomic classes are displayed for each functional group. Surface layers (SL), deep layers (DL) and deep chlorophyll maximum (DCM) are indicated.

In the Fig 3 Other Dinophyceae is a peculiar group because what are the other than Other Dinophyceae? It should be clear from the figure as stand-alone

**Response:** Functional groups have been renamed 'unclassified Dinophyceae' and 'trophy undetermined' throughout the manuscript to avoid confusion.

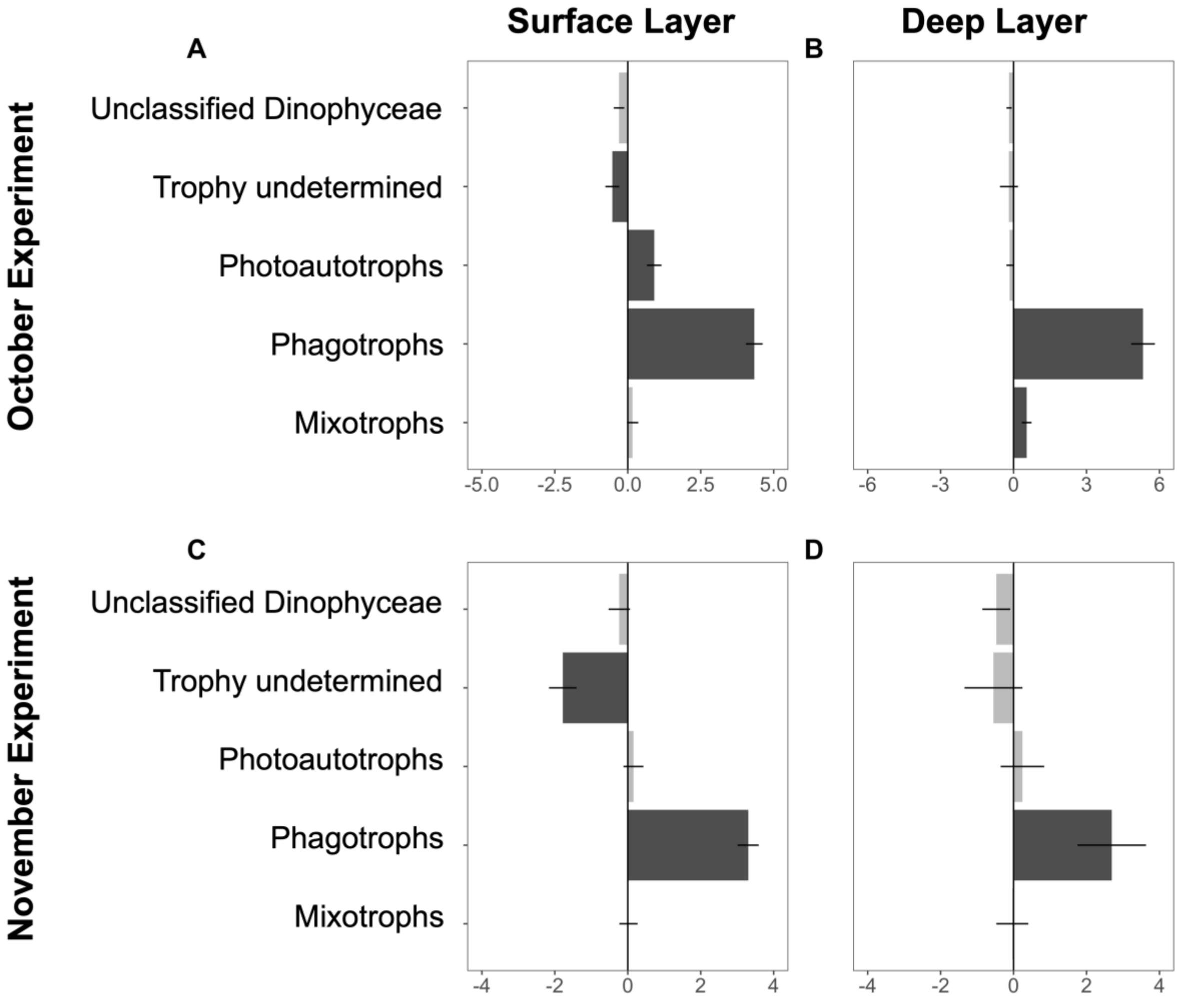


**Figure 4.** Magnitude of change in trophic groups between initials (t0) and controls for the month of October (A, B) and November (C, D). Magnitude of change is expressed in $log_2$ fold change, as estimated by the DESeq2 analysis. Dark bars represent groups for which the change was statistically significant (*p*-values < 0.05, two-sided Wald test corrected with the Benjamini & Hochberg method). Horizontal lines show the standard error.

Fig. 4: Since figures are stand-alone features, I suggest writing in the Figure legend the meaning of the abbreviations. I would write “ASV diversity” above the left panel and “Mode of Nutrition“ above the right.

**Response:** We thank Reviewer 1 for their suggestion and have made the changes accordingly, see below.

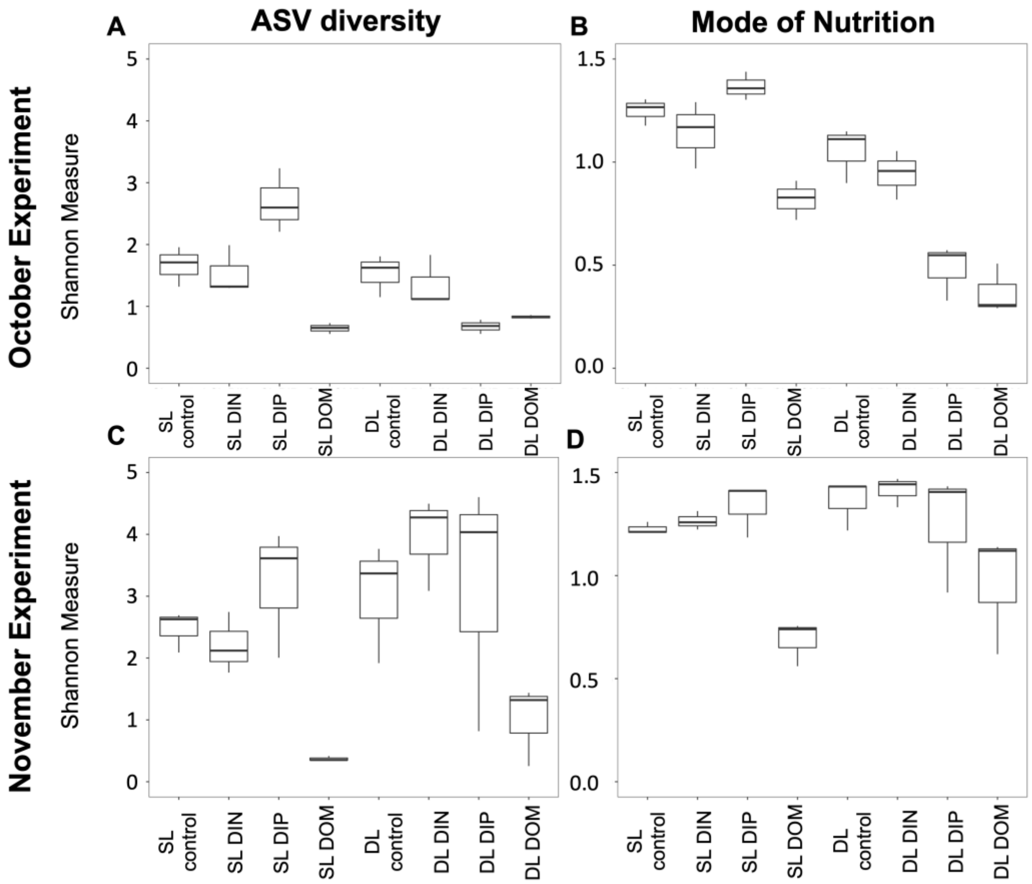


**Figure 6.** Comparison of Shannon measures of diversity between the different experimental treatments (controls; dissolved inorganic nitrogen, DIN; dissolved inorganic phosphorus, DIP; dissolved organic matter, DOM) by ASV diversity (A, C) and mode of nutrition (B, D) for October (A, B) and November (C, D) 2020 in both the surface layer (SL) and the deep layer (DL).

Fig. 5: replace Taxonomy with Taxonomic composition and Trophy with Nutritional mode. Meaning of abbreviations see Fig. 4.

**Response:** Changes have been made to the figure, see below.

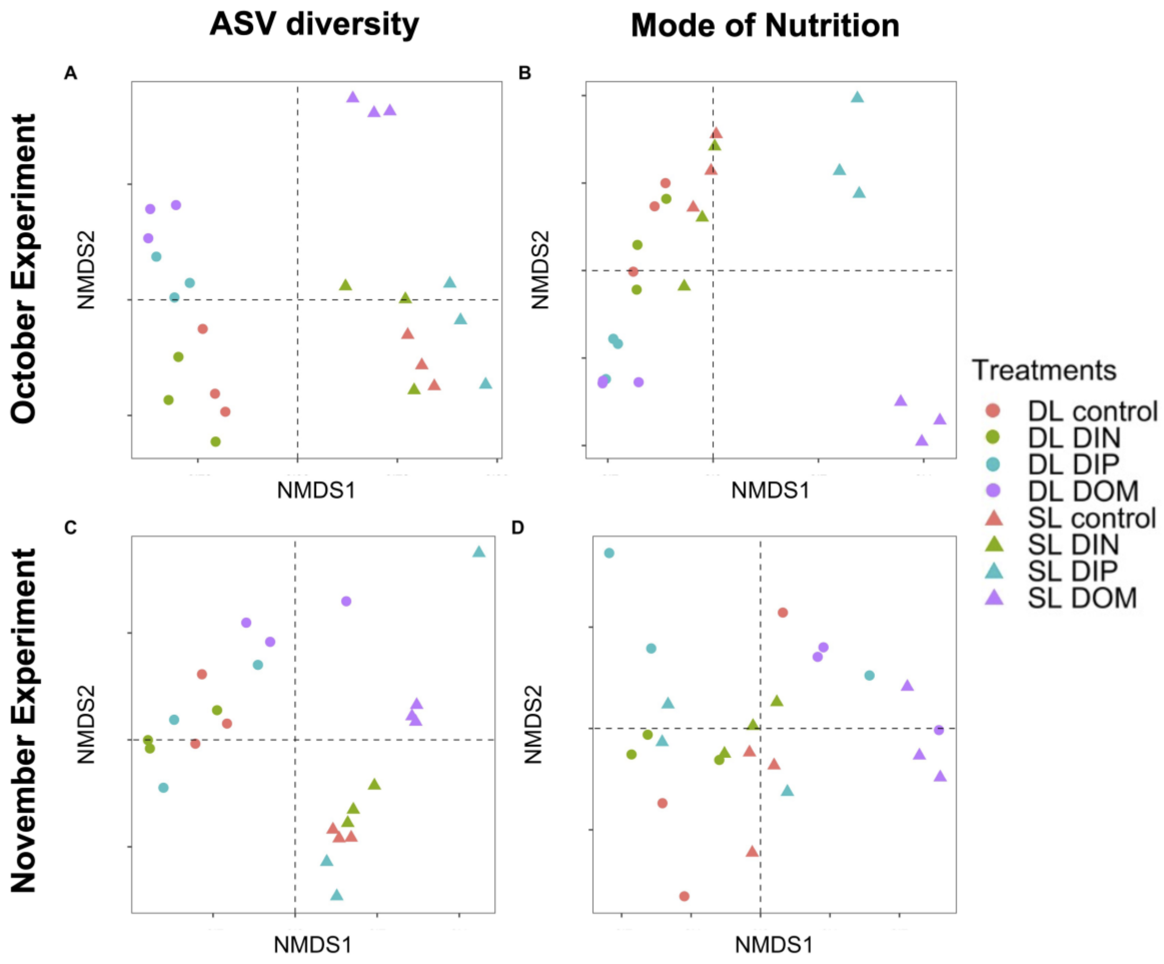


**Figure 7.** Non-metric multidimensional scaling (nMDS) ordinations of sample composition in the different experimental treatments (controls; dissolved inorganic nitrogen, DIN; dissolved inorganic phosphorus, DIP; dissolved organic matter, DOM) in terms of taxonomic composition (A, C) and nutritional mode (B, D) for October (A, B) and November (C, D) 2020 in both the surface layer (SL) and the deep layer (DL). 2-D stress indicated good fits (Kruskal, 1964) in all nMDS ordinations, with values less than 0.1 (A, 0.04; B, 0.02; C, 0.06; D, 0.01).

Fig. 6. A way to rebut the critique about the bottle effects could be to show a few heat maps of three replicates. If they show more or less the same relative abundances of taxonomic groups then that's it. Consider adding Surface layer and Deep layer on the left and right panel. This can be had from the indication below but it focuses the reader.

**Response:** We thank Reviewer 1 for their suggestion and have made the changes accordingly, see Figure 8. We also now provide heatmaps of the replicates for the study of the bottle effects in the Supplementary material in addition to Tables S5 and S6, see below Figure S2. These modifications to Figure 6, and the new figures in the Supplementary Material, highlight that differences between treatment replicates are much lower than the differences between treatments.

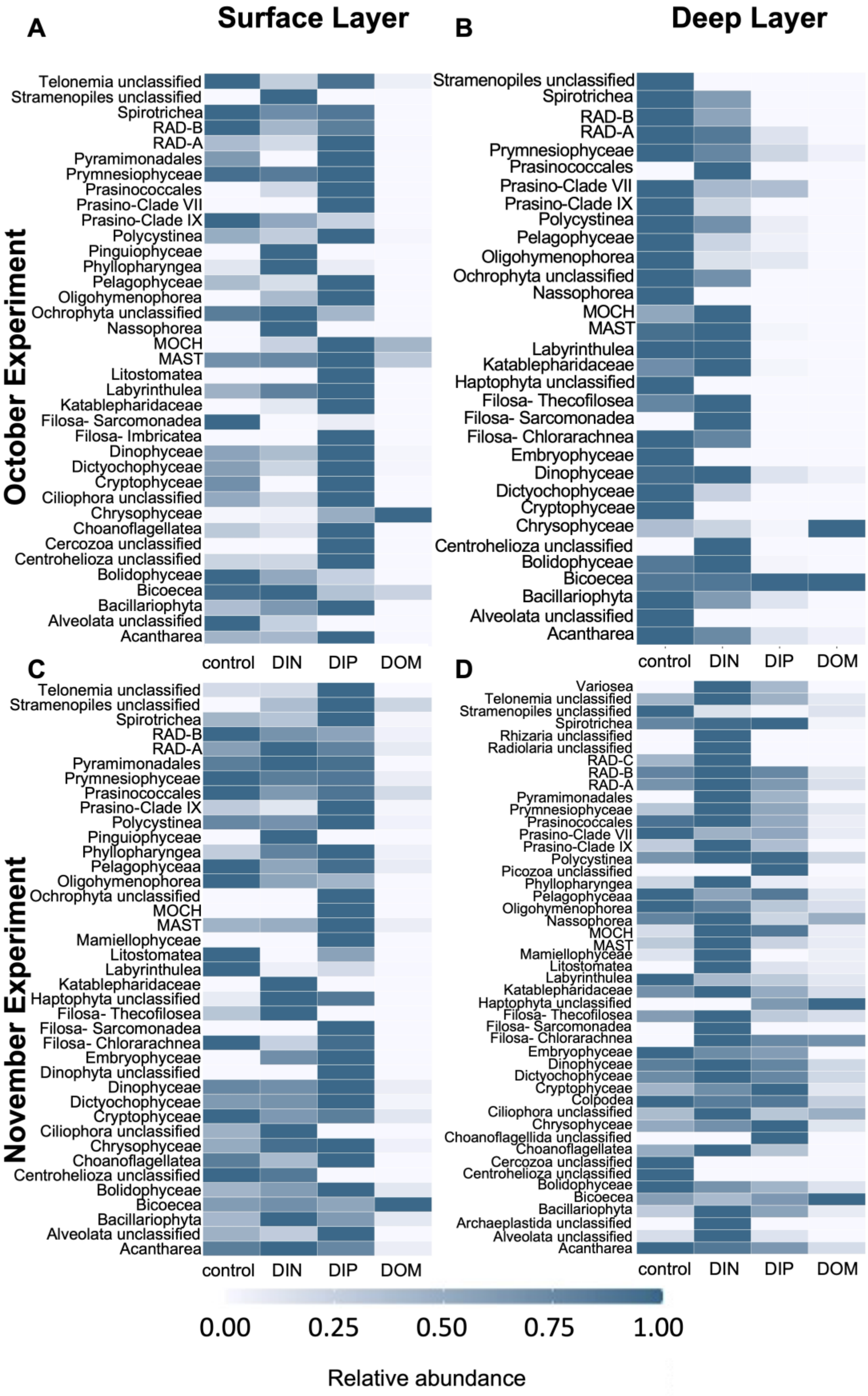
A
Surface Layer
B
Deep Layer
October Experiment
Telonemia unclassified
Stramenopiles unclassified
Spirotrichea
RAD-B
RAD-A
Pyramimonadales
Prymnesiophyceae
Prasinococcales
Prasino-Clade VII
Prasino-Clade IX
Polycystinea
Pinguiophyceae
Phyllopharyngea
Pelagophyceae
Oligohymenophorea
Ochrophyta unclassified
Nassophorea
MOCH
MAST
Litostomatea
Labyrinthulea
Katablepharidaceae
Filosa- Sarcomonadea
Filosa- Imbricatea
Dinophyceae
Dictyochophyceae
Cryptophyceae
Ciliophora unclassified
Chrysophyceae
Choanoflagellatea
Cercozoa unclassified
Centrohelioza unclassified
Bolidophyceae
Bicoecea
Bacillariophyta
Alveolata unclassified
Acantharea
Stramenopiles unclassified
Spirotrichea
RAD-B
RAD-A
Prymnesiophyceae
Prasinococcales
Prasino-Clade VII
Prasino-Clade IX
Polycystinea
Pelagophyceae
Oligohymenophorea
Ochrophyta unclassified
Nassophorea
MOCH
MAST
Labyrinthulea
Katablepharidaceae
Haptophyta unclassified
Filosa- Thecofilosea
Filosa- Sarcomonadea
Filosa- Chlorarachnea
Embryophyceae
Dinophyceae
Dictyochophyceae
Cryptophyceae
Chrysophyceae
Centrohelioza unclassified
Bolidophyceae
Bicoecea
Bacillariophyta
Alveolata unclassified
Acantharea
control DIN DIP DOM
control DIN DIP DOM
C
D
November Experiment
Telonemia unclassified
Stramenopiles unclassified
Spirotrichea
RAD-B
RAD-A
Pyramimonadales
Prymnesiophyceae
Prasinococcales
Prasino-Clade IX
Polycystinea
Pinguiophyceae
Phyllopharyngea
Pelagophyceaa
Oligohymenophorea
Ochrophyta unclassified
MOCH
MAST
Mamiellophyceae
Litostomatea
Labyrinthulea
Katablepharidaceae
Haptophyta unclassified
Filosa- Thecofilosea
Filosa- Sarcomonadea
Filosa- Chlorarachnea
Embryophyceae
Dinophyta unclassified
Dinophyceae
Dictyochophyceae
Cryptophyceae
Ciliophora unclassified
Chrysophyceae
Choanoflagellatea
Centrohelioza unclassified
Bolidophyceae
Bicoecea
Bacillariophyta
Alveolata unclassified
Acantharea
Variosea
Telonemia unclassified
Stramenopiles unclassified
Spirotrichea
Rhizaria unclassified
Radiolaria unclassified
RAD-C
RAD-B
RAD-A
Pyramimonadales
Prymnesiophyceae
Prasinococcales
Prasino-Clade VII
Prasino-Clade IX
Polycystinea
Picozoa unclassified
Phyllopharyngea
Pelagophyceaa
Oligohymenophorea
Nassophorea
MOCH
MAST
Mamiellophyceae
Litostomatea
Labyrinthulea
Katablepharidaceae
Haptophyta unclassified
Filosa- Thecofilosea
Filosa- Sarcomonadea
Filosa- Chlorarachnea
Embryophyceae
Dinophyceae
Dictyochophyceae
Cryptophyceae
Colpodea
Ciliophora unclassified
Chrysophyceae
Choanoflagellida unclassified
Choanoflagellatea
Cercozoa unclassified
Centrohelioza unclassified
Bolidophyceae
Bicoecea
Bacillariophyta
Archaeplastida unclassified
Alveolata unclassified
Acantharea
control DIN DIP DOM
control DIN DIP DOM
0.00 0.25 0.50 0.75 1.00
Relative abundance

**Figure 8.** Comparison of the relative abundances of each taxonomic class between treatments (controls; dissolved inorganic nitrogen, DIN; dissolved inorganic phosphorus, DIP; dissolved organic matter, DOM) in communities sampled both at the surface layer (A, C) and deep layer (B, D) for the experiment of October (A, B) and the experiment of November (C, D) 2020. Saturated blue tiles corresponded to the treatments with the highest relative abundances of each class while white tiles corresponded to the treatment with the lowest relative abundance. Heatmaps showing the relative abundances for the individual replicates of the November experiment are available in Figure S3.

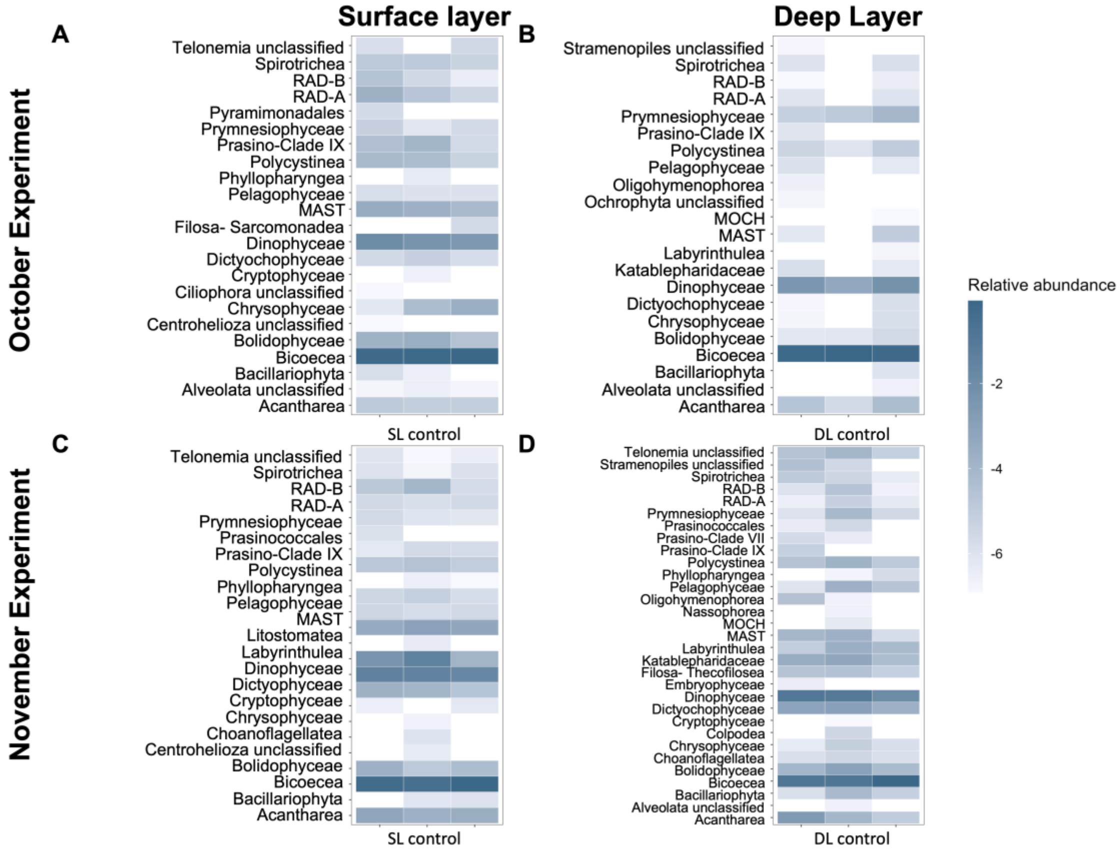


**Figure S3.** Comparison of the relative abundances of each taxonomic class between replicates of control treatments for the experiment of October (A, B) and the experiment of November (C, D) for both the surface layers (SL; A, C) and deep layers (DL; B, D). Saturated blue tiles corresponded to the treatments with the highest log value of relative abundances of each class while white tiles corresponded to the treatment with the lowest log value of relative abundance.

Table S2 DOM?

**Response:** We thank Reviewer 1 for pointing this out and apologise for the mistake. The table is now updated, see below.

| **Table S2.** Treatments of the nutrient enrichment assay and their different sources of nitrogen and phosphorus (dissolved inorganic nitrogen, DIN; dissolved inorganic phosphorus, DIP; dissolved organic matter, DOM) | |
|---|---|
| Treatments | Components |
| Control | no addition |
| **DIP** | $NaH_2PO_4$ |
| **DIN** | $NaNO_3$; $NH_4^+$ |
| **DOM** | |
| dissolved organic phosphorus | NAD; Phosphomonoester glycerophosphate; AMP; ATP |
| dissolved organic nitrogen | non-polar amino acids L-glycine, L-alanine and L-proline; polar amino acid L-serine, acidic amino acid L-glutamic acid; and urea |

**Reviewer 2:**
Comments to the Author
In "Organic and inorganic nutrients modulate taxonomic diversity and trophic strategies of small eukaryotes in oligotrophic oceans" Villiot et al. examined the phylogenetic and trophic structure of protist communities in the Sargasso sea linked to nutrient availability.
I personally found the nutrient enrichment assay coupled with 18S metabarcoding + Flow cytometry approach (for procaryotes) interesting. The manuscript well written and thoroughly documented. The conclusions are supported by the data, and the information is presented in a clear, credible way. The discussion is thorough and well composed, although reference list needs to be rechecked (i.e: use more contemporary publications). I really appreciated that authors provided supplementary information.
The paper fits fine to the scope of FEMS Microbes, however some minor revision can be performed prior its publication. I have general comments regarding the study.

**Comment:** We would like to thank Reviewer 2 for their review of our manuscript and valuable recommendations on how to improve the paper.

Abstract (general)
Need more info about the methods used to tackle the scientific question. There are both a field and laboratory works, right? Make it clear for the reader. Conclusions are general here

**Response:** Information on the method used in our study is now presented in the abstract. Lns 23-25: '*The study was conducted by means of field sampling of natural microbial communities and laboratory incubation of these communities under different nutrient regimes.*'

Introduction
- Introduction is too long. Hereby, I suggest few paragraphs to be removed.
- There is lacking an introductory info on the methods used.
- The predictions examined are fine. I suggest you re-structure the introduction focusing one by one on each of the predictions (in order), so that the reader gets to this point and has been introduced to each of the 3 points.

**Response:** We addressed Reviewer 2's comments by shortening the introduction as suggested and re-structuring the introduction for each prediction examined.

Lns 87-168: '*This study examines the following predictions of resource competition: 1) changes in inorganic nutrients, particularly nitrogen (N), will lead to significant changes in community taxonomic and trophic composition, 2) labile dissolved organic N (DON) and dissolved organic phosphorus (DOP) availability will benefit photosynthetic protist growth, and 3) increased nutrient availability will lead to the dominance of a limited number of taxa.*

*N and phosphorus (P) are the primary limiting nutrients for the growth of photosynthetic protists, with N and P availability regulating primary production and community structure (Moore et al., 2013). In fact, protists and other marine microbes are the main drivers of the global cycling of carbon, N and P in the ocean (Arrigo, 2005), through N and P uptake during growth and their loss to the dissolved phase through mortality or to depth via the biological carbon pump (Lalli and Parsons, 1997).*

*Studies demonstrating N limitation of protist growth in the oligotrophic ocean are numerous (e.g., Ryther and Dunstan, 1971; Mills et al., 2004), with phytoplankton biomass and productivity often increased only after the addition of N (e.g., Graziano et al., 1996; Moore et al., 2008). Due to the low atmospheric input of P into the ocean (Broecker and Peng, 1982), P may be an additional nutrient controlling primary production and the biological carbon pump (Moore et al., 2013). Evidence of P stress has been reported in microbial communities, with studies emphasizing P as a limiting nutrient*

*on physiologically-relevant time scales in the oligotrophic North Atlantic (e.g., Wu et al., 2000; Lomas et al., 2004; Van Mooy et al., 2009) and eastern Mediterranean (e.g., Thingstad et al., 2005).*

*Furthermore, although nutrient cycling studies often focus on inorganic N and P, in oligotrophic waters DOP may account for up to ~ 80 % of total dissolved P (Ruttenberg and Dyhrman, 2005) and DON may account for up to ~ 65 % of total dissolved N (Bronk, 2002). The remineralisation of DON and DOP in oligotrophic waters is thought to be mainly by heterotrophic microbes (Berman and Bronk, 2003), however, the use of DON and DOP by photosynthetic protists has also been widely reported (e.g., Antia et al., 1991; Mulholland and Lomas, 2008; Moschonas et al., 2017), and allows such primary producers to adapt to inorganic P limitation and support their continued growth (Fitzsimons et al., 2020).*'

L46: Please, check contemporary references i.e : Flynn et al. 2019, Leles et al., 2019.

**Response:** We have read the references suggested by Reviewer 2 and have added those we found relevant to the study into the introduction. Lns 48-56: '*Mixotrophic taxa in each of these protist groups (Havskum and Riemman, 1996) have the ability to live as both photoautotrophic primary producers and as heterotrophic consumers through ingestion and digestion of organic particles (Sanders, 1991). In the last decade, mixotrophy has been recognised as an important nutritional route for plankton communities (Leles et al., 2019; Schneider et al., 2020). For example, mixotrophic flagellate grazing on bacteria can contribute 50 to 95 % of bacterial mortality (Unrein et al., 2007; Zubkov and Tarran, 2008; Anderson et al., 2018), and significantly impact trophic transfer efficiency and the biological carbon pump (Ward and Follows, 2016).*'

The following refences were added to the list of literature:
Leles, S. G., Mitra, A., Flynn, K. J., Tillman, U., Stoecker, D., Jeong, H. J., Burkholder, J., Hansen, P. J., *et al.* (2019) Sampling bias misrepresents the biogeographical significance of constitutive mixotrophs across global oceans. Global Ecol. Biogeogr., 28, 418–428.
Schneider, L. K., Flynn, K. J., Herman, P. M. J., Troost, T. A. and Stolte, W. (2020) Exploring the trophic spectrum: placing mixoplankton into marine protist communities of the Southern North Sea. Front. Mar. Sci., 7, 586915.

L55 : specify « some » dinoflagellates

**Response:** We have changed this sentence to '*In addition, heterotrophic eukaryotes (in this study phagotrophs) such as ciliates and some dinoflagellates, are key component of food-webs as they act as important microbial predators consuming bacteria and small protists, and ultimately are preyed upon by mesozooplankton (Sherr and Sherr, 2002; Sherr et al., 2007).*' (Lns 61-64)

L71-L74: Remove this paragraph from the intro: "In oligotrophic systems, stratification is expected to strengthen with global warming (Sallée et al., 2021), further preventing deep nutrients from entering the surface layer,strengthening nutrient limitation and reducing primary production (Serret et al., 2015; Behrenfeld et al., 2006)."

**Response:** This paragraph was removed as well as the literature associated.

L101-L110: Remove this paragraph from the intro: "Protist metabolic activity and exudation influences the chemical composition of dissolved organic molecules (Kujawinski et al., 2016), with the metabolite production of photosynthetic microbes contributing up to 40% of total (dissolved and particulate) net primary production in oligotrophic systems (López-Sandoval et al., 2011). In return, dissolved organic matter composition (DON, DOP) and fluxes shift metabolic capabilities of marine microbes, and mediate the relationships between species (Kujawinski et al., 2016). Exploring which protist groups respond to different resources, and assessing the consequences for ecosystem function,

are a research priority as environmental change moderates the interaction(s) between the abiotic environment and the biotic community interactions."

**Response:** This paragraph was removed as well as the literature associated.

L124: Either remove this sentence or be more informative: "We examined the phylogenetic and trophic structure of protist communities in the Sargasso Sea during the fall of 2020".

**Response:** We modified the sentence and added the method used. Lns 185-189: '*In this field study, we examined the phylogenetic and trophic structure of protist communities in the Sargasso Sea during the fall of 2020 as well as the changes in composition triggered by addition of nutrients. Natural plankton communities were sampled and incubated during a nutrient enrichment assay to assess the effect of inorganic and organic nutrient availability on protist taxonomic and functional composition by means of 18S V4 DNA-metabarcoding.*'

Methods

L162: rare meso-zooplankton? Define it, please.

**Response:** Our experimental design excluded large meso-zooplankton which are significantly less abundant (rare) (<0.4 individuals $L^{-1}$; *Maas et al*., 2021) than pico-eukaryotes due to the low incubation volumes (2.2 L) used in our study. We now define large meso-plankton as plankton of size > 200 µm. Lns 249-252: '*The working volume of the incubation bottles was 2.2 L. Incubation bottles were filled with seawater from Hydrostation 'S' passed through a 200 µm mesh to exclude large meso-zooplankton (> 200 µm), whose low abundance (~ 0.15-0.35 individuals $L^{-1}$; Maas et al., 2021) would make their inclusion statistically rare.*'

The following reference was added:
Maas, A.E, Gossner, H., Smith, M. J. and Blanco-Bercial, L. (2021) Use of optical imaging datasets to assess biogeochemical contributions of the mesozooplankton. J. Plankton Res., 43, 475–491.

L237: How has this standardization been performed? Rarefraction? Please check Gloor et al. 2017. Metabarcoding data is compositional.

**Response:** We thank Reviewer 2 for pointing this out and apologise for our previous omission. Rarefaction was indeed applied to the dataset, we have now emphasized this point in the methods section, Lns 335-338: '*After excluding metazoan and "Eukaryota_unclassified" and standardization through rarefaction of all samples to 50,000 reads, all amplicon sequence variants (ASVs) with less than two reads (equivalent to global singletons) were equal to zero.*'

We agree with Reviewer 2 that recent papers suggest that standardization by rarefaction may be controversial in the case of microbial compositional datasets. Such studies recommend instead the use of centre log-ratio transformations, negating the need to perform rarefaction. This is based on the claim that scale invariant distances ensure equivalence between distances computed from absolute and relative abundance measurements. However, a **comparison** of Aitchison distances calculated using the robust CLR as well as the traditional CLR (calculated on a count matrix where zero values are imputed) **with** non-rarefied, non-transformed Euclidean distances and to rarefied, non-transformed Euclidean distances by P. Schloss (https://www.youtube.com/watch?v=ulo7WatBEAo&ab_channel=RiffomonasProject, Mars 2022) demonstrated that these log-ratios **do not** significantly improve the sensitivity / unevenness of the sampling effort. As a result of Schloss' conclusions, and the comment by Reviewer 3 that rarefaction is an essential step, we believe that rarefaction remains the most suitable standardization method for our study.

Results
L491: Result, not conclusion

**Response:** Sentence changed to '*To summarise, outputs of the examination of the Shannon and β-diversities as well as the taxonomic and trophic compositions were in good agreement with each other.*' (Lns 813-814)

Discussion
L635: Is there a difference between photo- and mixotrophs? Why do the authors think is not associated with the decline in photoautotrophs and specially mixotrophs?

**Response:** In our study we defined photoautotrophs as strict phototrophic eukaryotes while mixotrophs include taxa with '*the ability to live as both photoautotrophic primary producers and as heterotrophic consumers through ingestion and digestion of organic particles (Sanders, 1991).*' (Lns 49-51). The evidence based on the ASV detected (Table 1) and amplicon reads relative abundance by trophic groups (Fig. 4 and 5) did not show a significant decrease in either photoautotrophs or mixotrophs, rather there was an increase in phagotrophs, see Figure 4 below.

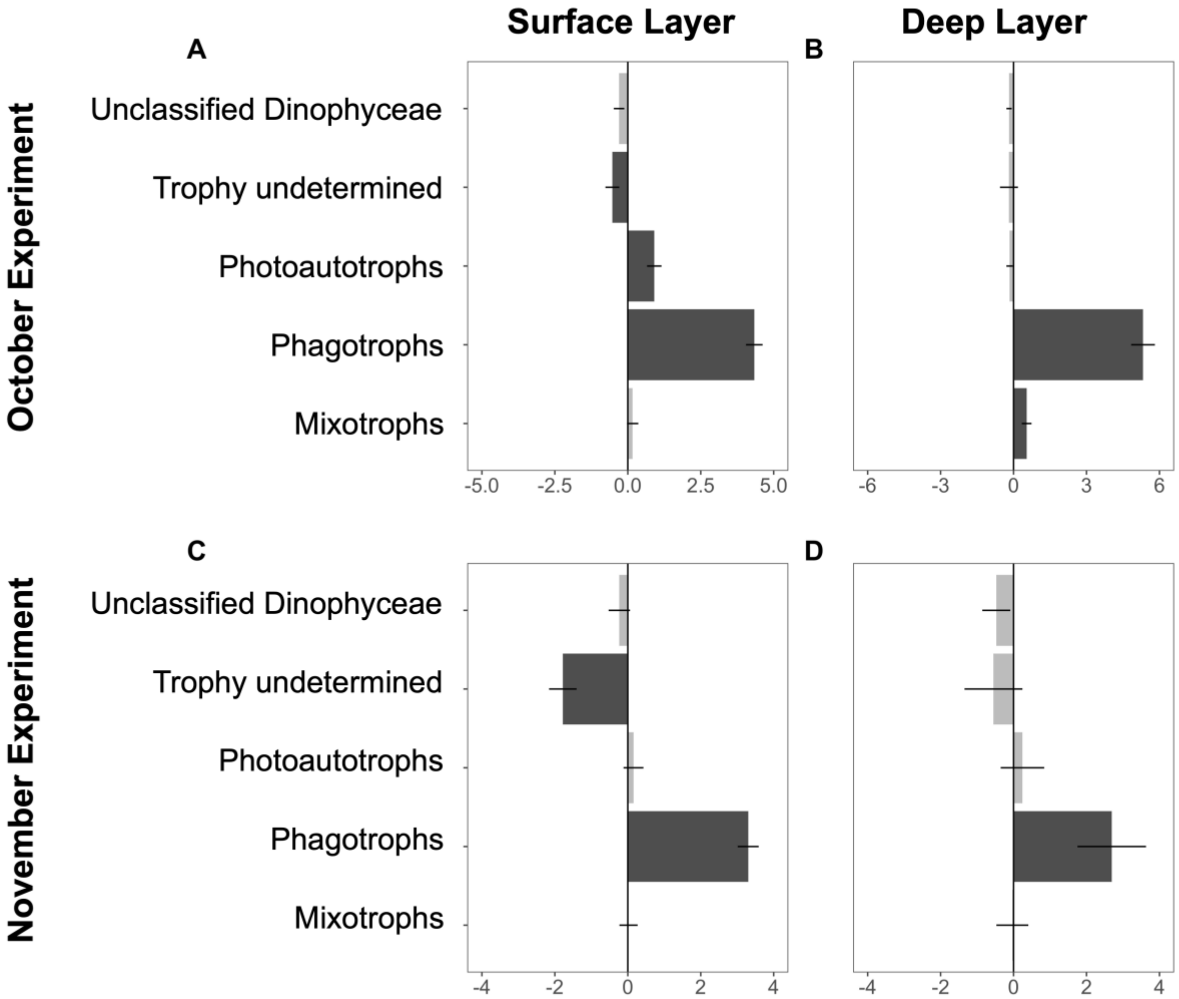


**Figure 4.** Magnitude of change in trophic groups between initials (t0) and controls for the month of October (A, B) and November (C, D). Magnitude of change is expressed in $log_2$ fold change, as estimated by the DESeq2 analysis. Dark bars represent groups for which the change was statistically significant (*p*-values < 0.05, two-sided Wald test corrected with the Benjamini & Hochberg method). Horizontal lines show the standard error.

L665: Check Lapeyra et al. 2022 for P limitation and changes in mixo- communities.

**Response:** We would like to thank Reviewer 2 for sharing this reference. We now make the parallel between our conclusions and those from Lapeyra Martin *et al*. (2022) in our Discussion: Lns 1154-1158: '*Similar observations were reported in a DNA-metabarcoding study of the mixoplankton community in the Belgian coastal zone, where inorganic P availability was the most important factor driving diversity and species richness of mixotrophs, autotrophs and heterotrophs, particularly in fall and winter (Lapeyra Martin et al., 2022).*'

The following reference was added: Lapeyra Martin, J., John, U., Royer, C. and Gypens, N. (2022) Fantastic Beasts: Unfolding mixoplankton temporal variability in the Belgian coastal zone through DNA-metabarcoding. Front. Mar. Sci., 9, 786787.

**<u>Reviewer 3:</u>**
<u>Comments to the Author</u>
This manuscript examined the changes in microeukaryotic plankton communities under nutrient amendments in oligotrophic waters - and found community shifts under various nutrient additions, in particular under phosphate enrichment. Phosphate concentrations are extremely low in Sargasso Sea, therefore a distinct community change under phosphate addition is not surprising - however some highlights from this study came from inferring functional traits from taxonomic composition.

The presented data look quite interesting and can extend our prior knowledge of potential eukaryotic community shift in open ocean oligotrophic waters, However, I found it was quite difficult to understand the detailed results - mostly due to insufficiently presented figures and interrupted my understanding the manuscript. I also found contradictory result from the conclusion - see the detailed comment in Fig. 5. Therefore, I recommend for publication after major modification in figures and texts.

**Comment:** We would like to thank Reviewer 3 for their review of our manuscript and valuable recommendations on how to improve the paper.

Fig. 1 - extremely difficult to see, fonts and symbols are too small, lines are too thin, colors are not clear, and too many parameters are included.

**Response:** As suggested by Reviewer 3, we made the lines and points bigger. We also added a colour legend to make the figure more readable and removed the dissimilarity data, see below.

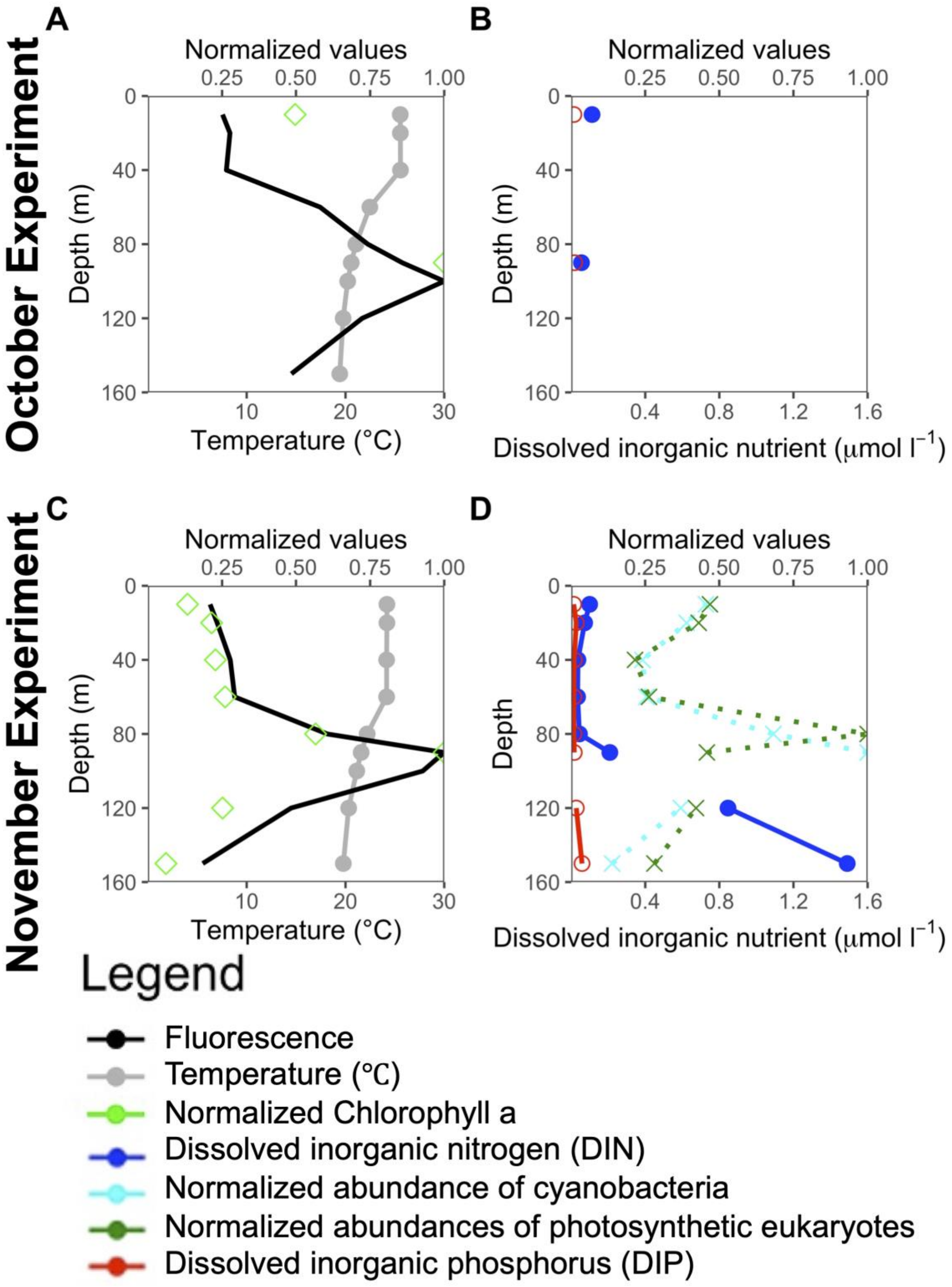


**Figure 1.** Vertical profiles of the physico-chemical and biological parameters at Hydrostation 'S' during sampling in October (A, B) and November (C, D). Temperature (grey dots) and fluorescence (solid black line) data correspond to *in situ* measurements recorded with a CTD. Normalized Chl-*a* (green diamonds), dissolved inorganic nitrogen (blue dots) and dissolved organic phosphorus (red dots), and normalized abundances of cyanobacteria (cross shape with dotted cyan line) and photosynthetic eukaryotes (cross shape with dotted green line) detected by flow cytometry (in November only), were all computed from samples collected with a rosette and processed in the laboratory.

For example, the Bray-Curtis dissimilarity should be presented in the separate figure - hard to see and no reason to include in this profile figure. The dissimilarity in community composition should be presented in more details in the separate figure using coordinate plots, like PCoA and both alpha and beta diversity should be included, in particular considering very similar community composition was observed in Figure 2. The associated texts for this result, lines 319-333 should be extended based on the new figures.

**Response:** We thank Reviewer 3 for their suggestion. We now present a new Figure 2 and the associated text in the result section, see below.

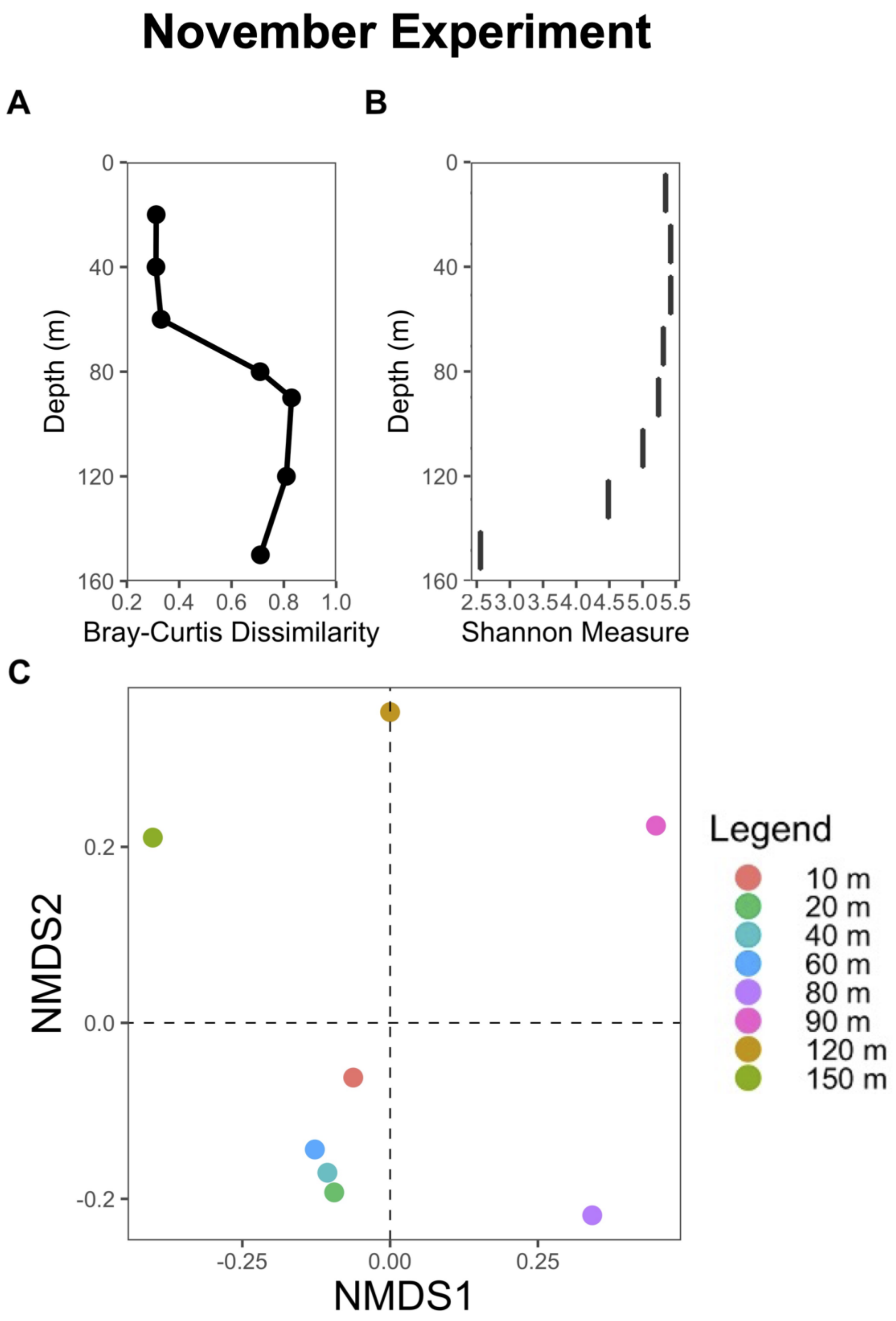


**Figure 2.** Analysis of the ASVs community composition along the depth gradient in November 2020: vertical profile of Bray-Curtis dissimilarity (A) and Shannon measures diversity (B), as well as non-metric multidimensional scaling (nMDS) ordination of the samples ASVs composition (C). 2-D stress indicated good fits (Kruskal, 1964) in the nMDS ordination, with a value less than 0.1.

To accompany the new and revised figures, we have added the following text:
Lns 463-465: '*The depths below (80 m, 90 m, 120 m, 150 m) revealed strong compositional changes when compared to the surface layer, with dissimilarity ranging from 71 to 83 % compared to 10 m (Fig. 2A, Supplementary Table S3).*'

Lns 486-494: '*Shannon diversity indices for each depth sampled in November further demonstrated relatively similar values within the mixed layer (~ 60 m), indicating communities of similar levels of diversity, and community differentiation below the deep layer (80 m) (Fig. 2B). Analysis of the β-diversities, based on Bray-Curtis dissimilarities of the relative abundances of ASVs, agreed with the assessment of Shannon diversity. The nMDS ordinations displayed noticeable differences between the clustering of samples from the mixed layer (i.e., depths 10 m; 20 m; 40 m; 60 m) and distinct clusters for each depth sampled below the deep layer, indicative of strong shifts in community ASVs (Fig. 2C).*'

Associated with this is whether rarefaction was done to allow for comparisons between samples with different sequencing depth. Rarefaction may be 'controversial' but is an essential step for testing the quality of the data. Is rarefaction performed? If not, provide it as a Supplementary Figure.

**Response:** Rarefaction was indeed applied to the dataset. We have now emphasized this point in the Methods section, Lns 335-338 '*After excluding metazoan and "Eukaryota_unclassified" and standardization through rarefaction of all samples to 50,000 reads, all amplicon sequence variants (ASVs) with less than two reads (equivalent to global singletons) were equal to zero.*'

Fig. 2 - panels A and B are almost useless since too many colors were used and not distinguishable - orders should be changed based on the taxonomy, not based on the alphabetical order. It does not make any sense.
I suggest make several panels based on the same taxonomy rank - e.g., chloroplastida, dinophyta, haptophyta, cryptophta, stramenopiles, etc.. otherwise no readers can really understand this result. Same for the panels C and D - include taxonomy data in addition to the functional traits so that readers can understand what proportion of taxonomy was attributed for each trait.

**Response:** We have now simplified the figure by adding taxonomic data (i.e., main phyla per trophic group) to the trophic composition**.** We have removed the taxonomic composition plots and instead provide this information in the **Supplementary material** in different panels for each phylum as suggested by the reviewer (see Figs. S1 and S2).

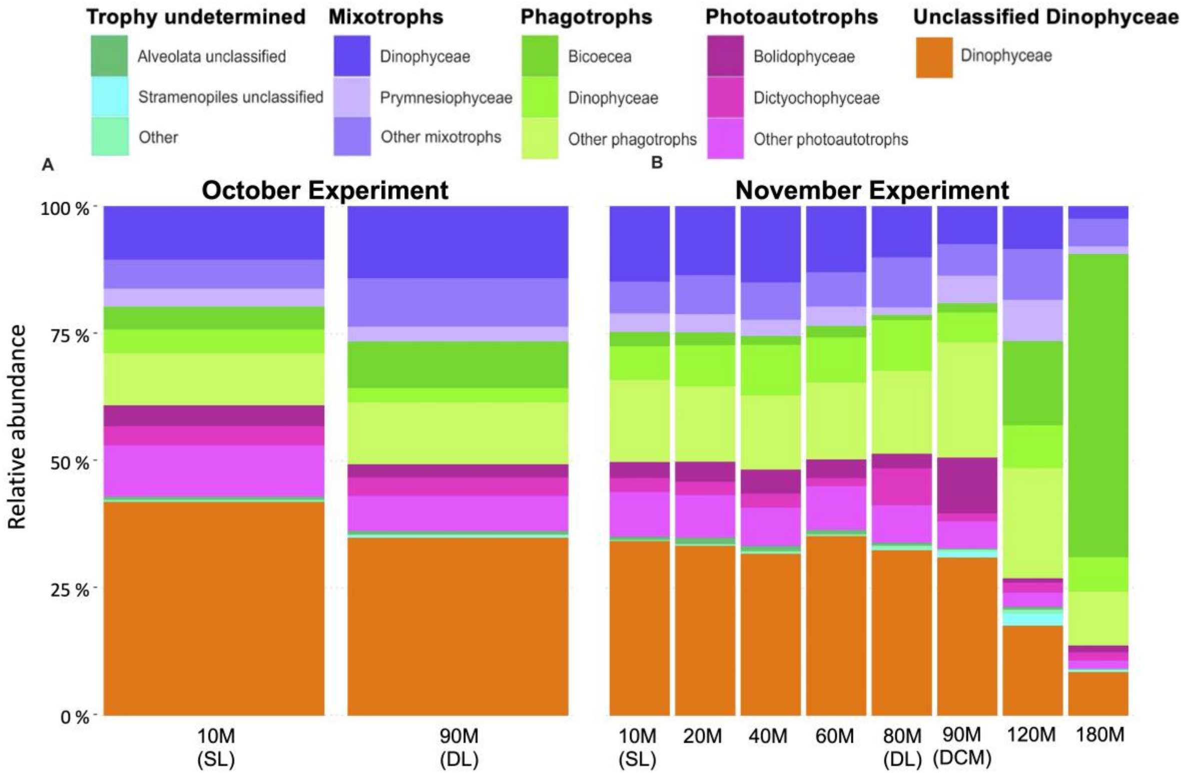


**Figure 3.** Taxonomic and functional composition (relative abundance, %) of the eukaryotic communities (excluding parasites and saprotrophs) in samples collected from different depths in the water column during October (A) and November (B). The two most abundant taxonomic classes are displayed for each functional group. Surface layers (SL), deep layers (DL) and deep chlorophyll maximum (DCM) are indicated.

And over 30% of reads were ‘other dino’ which is significant - since most dinoflagellates are known as heterotrophs or mixotrophs, I think it is worth to try testing results based on these two trophic scenarios and show whether it changes the result or not.

**Response:** While we agree with Reviewer 3 that 30% of reads is significant, as highlighted by Reviewer 1, these taxa tend to have higher copy numbers of 18S rDNA in their haploid genome than other lineages, leading to a bias in ASV and read abundances towards Dinophyceae. To account for this, we clustered the Dinophyceae ASVs which could not be classified as phagotrophs, mixotrophs or photoautotrophs in a separate functional group ‘unclassified Dinophyceae’. To address any potential further critique (and to acknowledge Reviewer 1’s comment), we now specifically mention this in the Methods section, Lns 365-403: ‘*Importantly, deviation inf the number of gene copies across plankton taxa (Not et al., 2009) can significantly affect the proportion of reads per taxa in complex microbial assemblages and lead to misinterpretation of relative abundances (Santi et al., 2021). This is particularly true in the case of Dinophyceae which tend to have a higher copy number of 18S rDNA than most lineages (Martin et al., 2022). Dinophyceae taxa made up a large proportion of our dataset, the Dinophyceae that could not be classified as phagotrophs, mixotrophs, or photoautotrophs (91.91 % of unclassified ASVs) were clustered as a separate functional group, ‘unclassified Dinophyceae*’

Authors should have ASV table that shows ASV abundance, taxonomy, and functional trait for each ASV - this is extremely useful for readers that want to understand trophic traits in details. Authors should prepare it as a Supplementary Data.

**Response:** The ASV reads table and associated functional traits (based on Adl *et al.*'s classification 2019) will be made available to the readers in the Supplementary material (see Table S8). In addition, bioproject (PRJNA849618) and biosamples are available on NCBI

Fig. 3 - I think Supplementary Fig. S1 is more informative than Fig. 3. So I suggest combine these two figures and make a new figure.

**Response:** Following Reviewer 3's recommendation, former Figure S1 is now presented in the main manuscript as Figure 5, see below. We did not combine the two figures for aesthetics reasons, but both figures are now discussed in the same result section to support our conclusions of the bottle effects.

Lns 640-647: '*Importantly, the ASV community composition were very similar between the natural community (t0) and after incubation (controls), with similar ASVs detected and relative abundances for the groups 'mixotrophs', 'photoautotrophs', and 'trophy undetermined, including 'unclassified Dinophyceae', while changes did occur for the phagotrophs (Figure 5). In fact, many phagotrophic classes were reduced or disappeared with incubation. In contrast, the class Bicoecea noticeably increased in all controls while other taxa (e.g., heterotrophic Dinophyceae, MAST, Telenomia, Spirotrichea, Litostomatea) were significantly reduced or absent from the samples after incubation (Fig. 5).*'

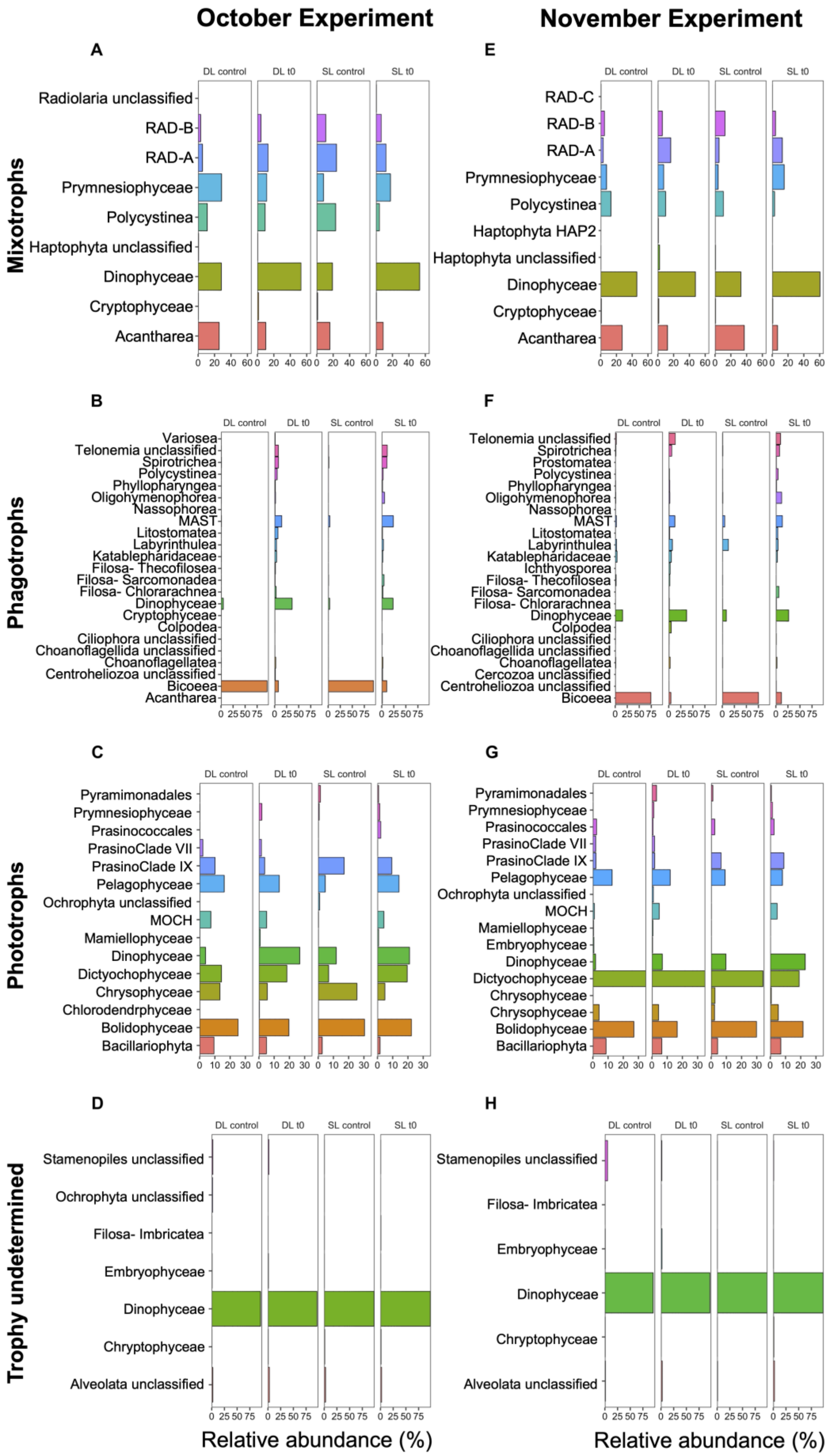
October Experiment
November Experiment
A
E
B
F
C
G
D
H
Mixotrophs
Phagotrophs
Phototrophs
Trophy undetermined
DL control
DL t0
SL control
SL t0
Radiolaria unclassified
RAD-B
RAD-A
Prymnesiophyceae
Polycystinea
Haptophyta unclassified
Dinophyceae
Cryptophyceae
Acantharea
RAD-C
Haptophyta HAP2
Variosea
Telonemia unclassified
Spirotrichea
Phyllopharyngea
Oligohymenophorea
Nassophorea
MAST
Litostomatea
Labyrinthulea
Katablepharidaceae
Filosa- Thecofilosea
Filosa- Sarcomonadea
Filosa- Chlorarachnea
Colpodea
Ciliophora unclassified
Choanoflagellida unclassified
Choanoflagellatea
Centroheliozoa unclassified
Bicoeea
Prostomatea
Ichthyosporea
Cercozoa unclassified
Pyramimonadales
Prasinococcales
PrasinoClade VII
PrasinoClade IX
Pelagophyceae
Ochrophyta unclassified
MOCH
Mamiellophyceae
Embryophyceae
Dictyochophyceae
Chrysophyceae
Chlorodendrphyceae
Bolidophyceae
Bacillariophyta
Stamenopiles unclassified
Filosa- Imbricatea
Chryptophyceae
Alveolata unclassified
Relative abundance (%)

**Figure 5.** Relative abundances of the taxonomic classes within the functional groups mixotrophs (A, E), phagotrophs (B, F), photoautotrophs (C, G), and 'trophy undetermined' including the 'unclassified Dynophyceae' (D, H) between sampling times (t0s) and controls from the surface layer (SL) and deep layer (DL) in October 2020 (A, B, C, D) and November (E, F, G, H) 2020.

Fig. 4 - change the y-axis has the same range, so that readers can properly compare the result.

**Response:** Y-axis have now been changed to allow comparison between the two 2 graphs of the ASV diversity and the 2 graphs of the Trophic diversity, see below.

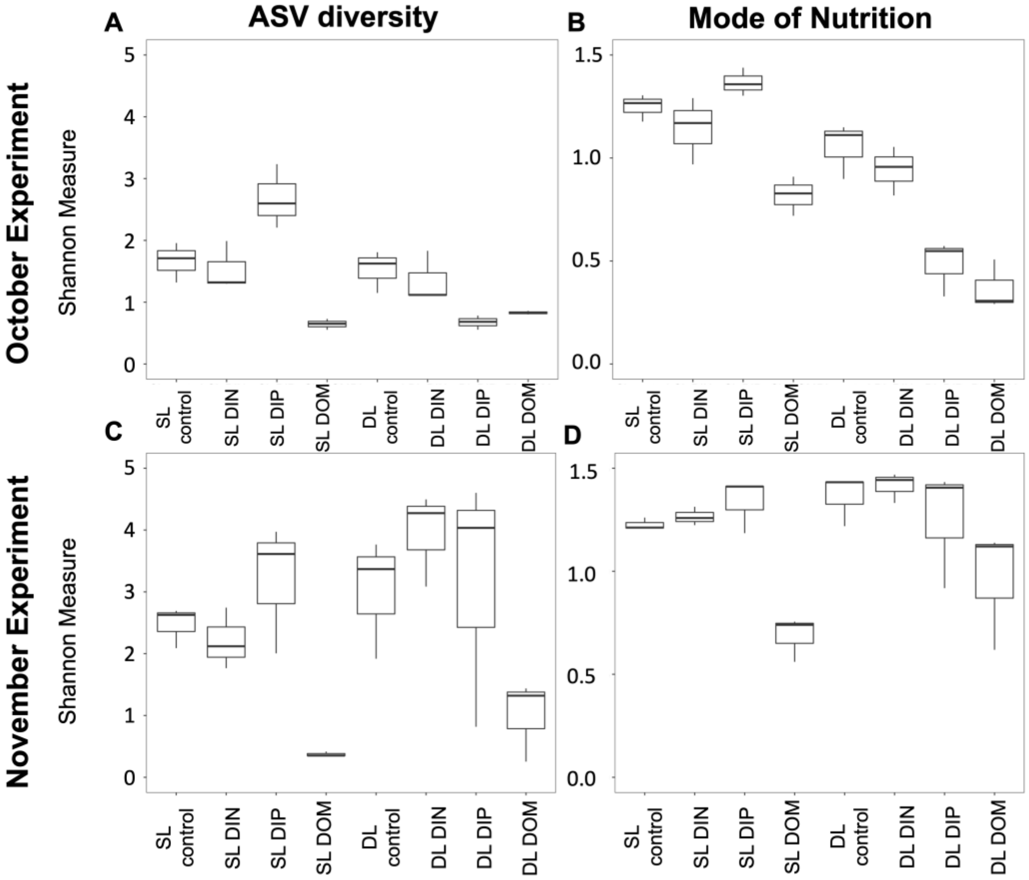


**Figure 6.** Comparison of Shannon measures of diversity between the different experimental treatments (controls; dissolved inorganic nitrogen, DIN; dissolved inorganic phosphorus, DIP; dissolved organic matter, DOM) by taxonomy (A, C) and mode of nutrition (B, D) for October (A, B) and November (C, D) 2020 in both the surface layer (SL) and the deep layer (DL).

Fig. 5 - author concluded that DIP addition induced distinct community changes, but I found this figure shows contradictory result. Both Fig. 5a and 5b show SL control, SL DIP, and SL DIN are all clustered together, and DL control is also clustered with DL DIP. Based on this coordinate plot, DIP did not change the community composition significantly. I suggest authors should reanalyze these results, and address it clearly in the text.

**Response:** Original text stipulates that in Fig. 5A (analysis of the taxonomic composition), the treatment DIP forms a distinct cluster from the control and DIN treatments **in the deep layer**, while SL DIP is clustered together with SL control and DIN. This is further supported by the analysis of alpha-diversity (Fig.4) with a significant *p*-value found for the comparison DL control and DL DIP (Dunn's test). The original text also stipulates that in Fig. 5B (analysis of the trophic composition) both DL DIP and SL DIP form distinct clusters from their respective controls and DIN treatments, see annotated figure below.

Original text: Lns 430-437 '*analysis of the species composition displayed strong taxonomic differences between the SL and the DL and revealed distinct clusters for* ***the treatment DIP*** *(Fig. 5A), which was* ***well separated from the control and DIN treatment clustered together in the DL****, while the* ***control as well as DIN and DIP treatments were clustered together for the SL****. The analysis of the trophic strategies identified* ***distinct clusters for DIP treatments from both the SL and DL (Fig. 5B)****. The SIMPER analysis identified a significant p-value (< 0.05) in the trophic group 'unclassified' for the comparison DL control vs DL DIP.*'

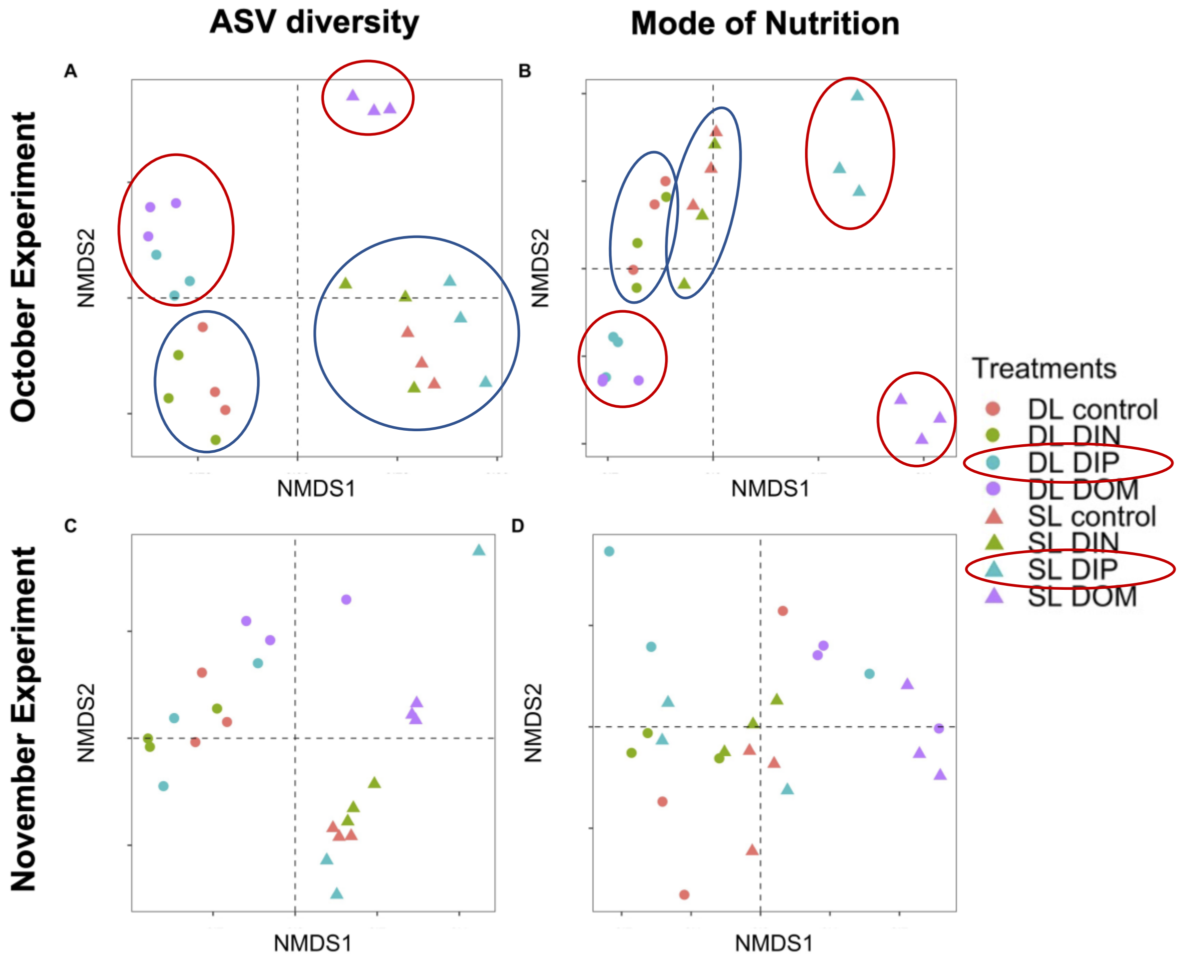


In this section 'Nutrient response if the protist community- (i) Diversity and composition', community compositions both in terms of **taxonomic compositions and trophic compositions** are compared between treatments supported by analysis of the alpha-diversity (i.e., Shannon measure) and subsequent Kruskal-Wallis rank sum test and Dunn's Kruskal-Walllis multiple comparison; as well as **beta-diversity** and associated ANOSIM, PERMANOVA and SIMPER. Because of the large set of information provided to the reader, we understand there may have been some confusion. However, we do not believe that contradictory results were presented from Figure 5 (now Figure 7). We apologise for the lack of clarity in presenting the results and we have now re-structured the section to make it easier for the reader to follow our reasoning, as well as modified Figure 5 to make the distinctions **SL vs DL** and **controls vs DIP** clearer. We hope this fully satisfies the reviewer's comments.

We have now re-structured the sentence above to add clarity and to try and avoid further confusion. See revised manuscript Lns 726-749: '*the analysis of the ASV composition displayed strong taxonomic differences between the SL and the DL. Analysis of the ASV composition revealed a distinct cluster for the treatment DIP (Fig. 7A) in the DL which was well separated from the control and DIN treatment clustered together (Fig. 7A). In the SL however, the control, DIN and DIP treatments were*

*clustered together (Fig. 7A). The analysis of the trophic strategies identified distinct clusters for DIP treatments from both the SL and DL (Fig. 7B). The SIMPER analysis identified a significant p-value (< 0.05) in the group 'trophy undetermined' for the comparison DL control vs DL DIP.'*

Fig. 6 - I think it is better to present like Figs 8 and 9, so the readers can easily compare these results.

**Response: We believe that the reviewer has misinterpreted our paper here.** We would like to emphasise that Figures 8 and 9 present outputs of differential abundances of the trophic groups where each treatment is compared to the community composition of the control (DIN versus control; DIP versus control; DOM versus control). In contrast, Figure 6 (Figure 8 in the new revised manuscript) presents a comparison of the taxonomic classes relative abundances within the protist community between all treatments (control versus DIN versus DIP versus DOM). As reported by Reviewer 1, a heatmap is a more robust comparison of relative abundances of taxonomic groups between multiple samples and therefore we believe that a heatmap is the most suitable way to present these outputs.

Fig. 7 - this is quite interesting data that needs to be addressed more details. Are there any data for T0 so readers can understand the initial condition of incubation experiments? And are these cyanos prochlorococcus or synechococcus? Better to add the details and should be discussed based on T0 data.

**Response:** We thank Reviewer 3 for pointing this out and have now added data for t0 to the figure, see below. Cyanobacteria include both *Prochlorococcus* and *Synechococcus* species, we now emphasise this point in the Figure Legend and methods section. We also briefly compare t0s and controls relative abundances presented in Figure 9.

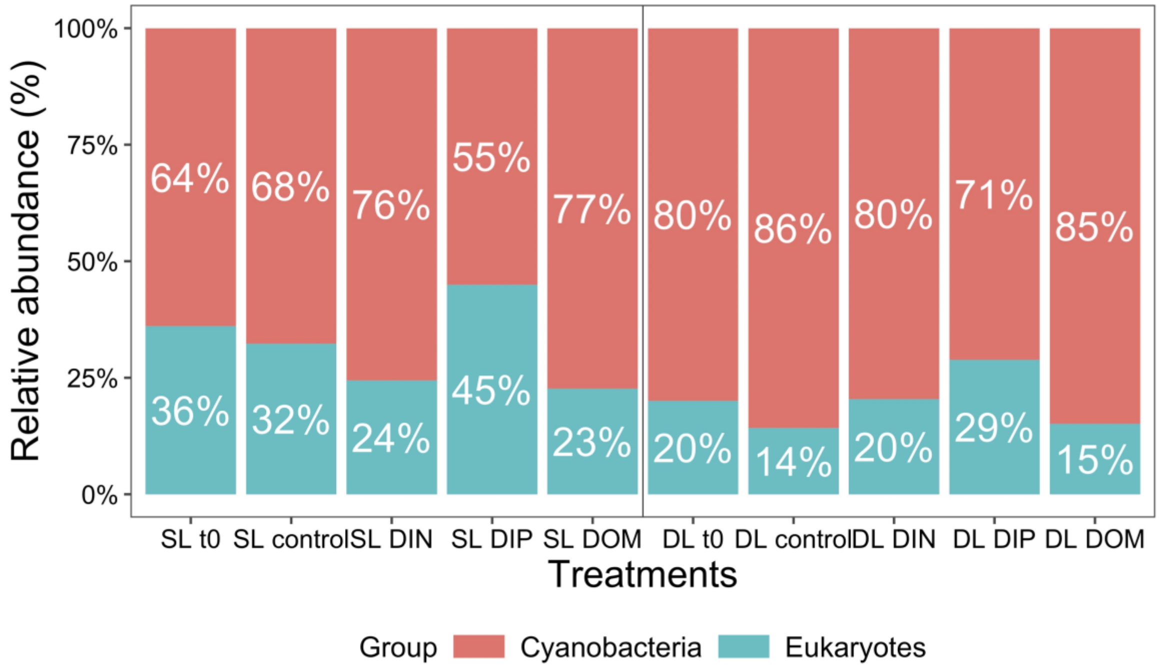


**Figure 9.** Relative abundances (%) of cyanobacteria, including *Synechococcus* and *Prochlorococcus*, and eukaryotes determined by flow cytometry (FL3/FSC) for each treatment (before incubation, t0; after incubation, controls; dissolved inorganic nitrogen, DIN; dissolved inorganic phosphorus, DIP; dissolved organic matter, DOM) in communities sampled both in the surface (SL) and deep layer (DL) in November 2020.

Lns 346-349: '*Cells were characterized by using scatter and fluorescence signals collected from the 488 nm laser as 'cyanobacteria' (including both Prochlorococcus and Synechococcus) and 'eukaryotes' (corresponding to photosynthetic eukaryotes with cell diameters > 2 μm).*'

Lns 851-855: *'In November, relative abundances of cell counts of photosynthetic eukaryotic (photoautotrophs and mixotrophs) and prokaryotic (cyanobacteria including Synechococcus and Prochlorococcus) particles detected by flow cytometry (likely cell diameters < 10 μm) showed similar profiles between samples before and after incubation (t0s versus controls) in both the SL and DL (Fig. 9). After incubation, normalised counts of particles indicated that the highest abundances of photosynthetic eukaryotes were in the DIP treatments for both layers (Table 3, see also Fig. 9).'*
